# Angiotensin II Is a New Component Involved in Splenic T Lymphocyte Responses during *Plasmodium berghei* ANKA Infection

**DOI:** 10.1371/journal.pone.0062999

**Published:** 2013-04-30

**Authors:** João Luiz Silva-Filho, Mariana Conceição Souza, Claudio Teixeira Ferreira-DaSilva, Leandro Souza Silva, Maria Fernanda Souza Costa, Tatiana Almeida Padua, Maria das Graças Henriques, Alexandre Morrot, Wilson Savino, Celso Caruso-Neves, Ana Acacia Sá Pinheiro

**Affiliations:** 1 Instituto de Biofísica Carlos Chagas Filho, Universidade Federal do Rio de Janeiro, Rio de Janeiro, RJ, Brazil; 2 Instituto de Tecnologia em Fármacos, Fundação Oswaldo Cruz, Rio de Janeiro, RJ, Brazil; 3 Instituto de Microbiologia Professor Paulo de Góes, Universidade Federal do Rio de Janeiro, Rio de Janeiro, RJ, Brazil; 4 Departamento de Imunologia, Fundação Oswaldo Cruz, Rio de Janeiro, RJ, Brazil; 5 Instituto Nacional de Ciência e Tecnologia em Biologia e Bioimagem, Conselho Nacional de Desenvolvimento Científico e Tecnológico/MCT, Rio de Janeiro, RJ, Brazil; 6 Instituto Nacional para Pesquisa Translacional em Saúde e Ambiente na Região Amazônica, Conselho Nacional de Desenvolvimento Científico e Tecnológico/MCT, Rio de Janeiro, RJ, Brazil; University of Sydney, Australia

## Abstract

The contribution of T cells in severe malaria pathogenesis has been described. Here, we provide evidence for the potential role of angiotensin II (Ang II) in modulating splenic T cell responses in a rodent model of cerebral malaria. T cell activation induced by infection, determined by 3 to 4-fold enhancemen**t** in CD69 expression, was reduced to control levels when mice were treated with 20 mg/kg losartan (IC_50_ = 0.966 mg/kg/d), an AT_1_ receptor antagonist, or captopril (IC_50_ = 1.940 mg/kg/d), an inhibitor of angiotensin-converting enzyme (ACE). Moreover, the production of interferon-γ and interleukin-17 by CD4^+^ T cells diminished 67% and 70%, respectively, by both treatments. Losartan reduced perforin expression in CD8^+^ T cells by 33% while captopril completely blocked it. The upregulation in chemokine receptor expression (CCR2 and CCR5) observed during infection was abolished and CD11a expression was partially reduced when mice were treated with drugs. T cells activated by *Plasmodium berghei* ANKA antigens showed 6-fold enhance in AT_1_ levels in comparison with naive cells. The upregulation of AT_1_ expression was reduced by losartan (80%) but not by captopril. Our results suggest that the AT_1_/Ang II axis has a role in the establishment of an efficient T cell response in the spleen and therefore could participate in a misbalanced parasite-induced T cell immune response during *P. berghei* ANKA infection.

## Introduction

Malaria is a life-threatening parasitic disease that infects more than 500 million people per year and kills more than 1 million [1]. Although host immunity acquired through repeated exposure to the pathogen can limit infection and control parasitemia, the immune reactions also contribute to pathogenesis and fatalities. A large body of work using the experimental model with *Plasmodium berghei* ANKA has provided a significant contribution to understanding the pathogenesis of malaria, including cerebral malaria (CM), one of the most severe complications of *Plasmodium falciparum* infection. The murine infection is described as similar to human disease in some relevant clinical and pathologic aspects [2], [3]. A lot of information about malaria pathogenesis is available in the literature; however, the precise mechanisms underlying malaria complications are not well defined. It is believed that severe malaria is caused by a combination of parasitic factors and high levels of proinflammatory cytokines such as tumor necrosis factor (TNF)-α, lymphotoxin-α, interferon (IFN)-γ [4]–[6] as well as different effector cells such as CD4^+^ T cells, CD8^+^ T cells, natural killer T cells, and natural killer cells [7]–[11]. The contribution of T cells to disease was initially described in neonatal thymectomized golden hamster, which did not develop the syndrome after infection with *P. berghei* [12] **.** Several other reports reinforced the specific roles of T cell subsets in the pathogenesis of CM [13], [14]. Moreover, the presence of leukocytes in the brain vasculature during infection suggests that intravascular infiltration of such cells could be associated with a local inflammatory response contributing to the induction of the disease [2], [15]. Thus, the signals and consequently the cellular and molecular mechanisms involved in T cell activation are crucial to understand the role of these cells during infection.

In recent years, new components involved in T cell activation have been reported. T lymphocytes display a functional renin–angiotensin system (RAS) able to produce and deliver angiotensin II (Ang II) to sites of inflammation [16]. In turn, Ang II acts as a co-stimulus for T cell activation *in vitro* [17], proliferation and production of IFN-γ, suggesting that it induces differentiation to the Th1 phenotype [16], [18]. Moreover, Ang II also has chemoattractant properties, because it induces transmigration of activated T cells on contact with laminin or fibronectin [17]. However, the potential role of RAS peptides, especially Ang II, in modulating T cell responses during malaria is poorly understood.

Based on the notion that an unbalanced cell-mediated immune response is involved in severe malaria, we thoroughly investigated whether Ang II could modulate the function of T cells derived from the spleen of mice infected with *Plasmodium berghei* ANKA. Our findings reveal that Ang II has a direct effect on both CD4^+^ and CD8^+^ T lymphocytes, inducing upregulation of different surface activation markers, cell differentiation, and enhancing adhesion/transmigration capacity. It is still not clear whether lymphocyte trafficking induced by Ang II affects the development of organ-specific inflammation and fatalities during malaria infection. The precise description of such mechanisms could represent an important elucidation of new components involved in the regulation of splenic T cell responses during *P. berghei* ANKA infection.

## Materials and Methods

### Ethics Statement

This work was carried out in strict accordance with the recommendations in the Guide for the Care and Use of Laboratory Animals of the National Institutes of Health. The protocol was approved by the Institutional Ethics Committee of Federal University of Rio de Janeiro (permit number CEUA-CCS-098) and by The Committee on Ethical Use of Laboratory Animals of Fundação Oswaldo Cruz (permit number L004/08).

### Mice and Parasites

C57BL/6 mice (8–12 weeks) were provided by Fundação Oswaldo Cruz Breeding Unit (Rio de Janeiro, Brazil) and bred at the Laboratory of Applied Pharmacology Experimental Animal Facility, Farmanguinhos (Fundação Oswaldo Cruz). The mice were caged with free access to food and fresh water in a temperature-controlled room (22–24°C) with a 12 h light/dark cycle until used. A cryopreserved sample of *Plasmodium berghei* ANKA-infected erythrocytes was kindly provided by Dr Leonardo J. Carvalho, La Jolla University, San Diego, CA. The sample was thawed and inoculated intraperitoneally into a naive C57BL/6 mouse. Cells were maintained in mice up to 9 passages prior to use.

Groups of 20 mice were infected i.p. with 5×10^6^ freshly passaged *P. berghei* ANKA parasitized erythrocytes and parasitemia was monitored by counting 10 microscope fields from Giemsa-stained thick blood smears every 2–3 days [19]. The percentage of parasitemia was described as the number of parasitized red blood cells in 100 erythrocytes. Mice infected with *P. berghei* ANKA were divided into 3 groups: vehicle-; losartan- or captopril-treated mice. The treatments began on the day of infection and were administered by gavage at a dose of 20 mg/kg per day for 6 days. Naive mice were used as controls. Mortality was checked daily. In all experiments, mice were euthanized on day 6 to isolate splenic or brain T cells and plasma. Cerebral malaria was determined as described previously [20]. Briefly, 6 days after infection, behavioral, motor and functional analyses were assessed by SHIRPA primary screen. The scoring system described by Martins et al. [20] was used and a score lower than 11 was considered positive to CM.

### Compounds

*N*-2-Hydroxyethylpiperazine *N*-2-ethanesulfonic acid (HEPES), (tris350 hydroxymethyl)-aminomethane (Tris), glucose, sodium bicarbonate, sodium azide, bovine serum albumin (BSA), PD123319, Ang II (Asp-Arg-Val-Tyr-Ile-His-Pro-Phe), were purchased from Sigma Aldrich Co. (St. Louis, MO, USA). The AT_1_ receptor selective antagonist, losartan, was obtained from Merck S.A. (Rio de Janeiro, RJ, Brazil) and captopril, the ACE inhibitor, was obtained from Laboratório Farmacêutico da Marinha (Rio de Janeiro, RJ, Brazil). Fluorescein isothiocyanate (FITC)-, phycoerythrin (PE)- and PE-Cy5.5-conjugated hamster IgG1 anti-murine CD3 (145-2C11), PE- and PerCP-conjugated rat IgG2a anti-murine CD4 (RM4-5), PE-conjugated rat IgG2a anti-murine CD8a (53–6.7), FITC-conjugated rat IgG2a anti-murine CD45R/B220 (RA3-6B2), PE-Cy5-conjugated rat IgG2b anti-murine CD44 (IM7), FITC-conjugated rat IgG2a anti-murine CD62L (MEL-14), PE-conjugated rat IgG1 anti-murine IFN-γ (XMG1.2), PE-conjugated rat IgG1 anti-murine IL-17 (TC11-18H10), PE-conjugated rat IgG2b anti-murine IL-10 (JES5-16E3), PE-conjugated rat IgG2b anti-murine IL-4 (BVD4-1D11), biotin-conjugated rat IgG2a anti-murine CD11a (MD17/4), PE-conjugated streptavidin, PerCP/PE/FITC-conjugated hamster IgG1 and IgG2, and goat IgG2a isotype controls were all purchased from BD Pharmingen (San Diego, CA, USA). FITC-conjugated rat IgG2b anti-murine CD4 (GK1.5), APC-conjugated rat IgG1 anti-murine CD25 (PC61.5), APC-conjugated hamster IgG anti-murine CD69 (H1.2F3), PE-conjugated rat IgG2a anti-murine Foxp3 (FJK-16s) were purchased from eBioscience (San Diego, CA, USA). Goat polyclonal IgG anti-murine CCR2, goat polyclonal anti-murine CCR5, goat polyclonal anti-murine perforin and FITC-conjugated goat IgG anti-murine IgG were obtained from Santa Cruz Biotechnology, Inc. (Santa Cruz, CA, USA). Dulbecco’s minimal essential medium (DMEM) high glucose, RPMI 1640, fetal bovine serum (FBS), ACK lysing buffer, minimal essential medium (MEM), nonessential amino acid solution (MEM AAs), 2-mercaptoethanol, l-glutamine, sodium pyruvate, penicillin/streptomycin and trypan blue were obtained from GIBCO (Grand Island, NY, USA). Human fibronectin was purchased from Chemicon International Inc. (Temecula, CA, USA), human laminin and Alexa Fluor 488-phalloidin were purchased from Invitrogen (Eugene, OR, USA). All other reagents were of the highest purity available.

### Splenic T Cell Isolation and Culture

Mouse spleens were aseptically removed from each experimental group and placed in DMEM supplemented with 2 mM l-glutamine, 10 mM nonessential amino acids, 0.05 mM 2-mercaptoethanol, 1 mM sodium pyruvate, 100 U/ml penicillin, 100 U/ml streptomycin and 10 mM HEPES. Single cell suspensions from the spleens were treated with ACK solution to induce lysis of erythrocytes and washed twice with Hanks’ solution supplemented with 10% FBS. Cells were resuspended in complete medium (DMEM with 10% FBS), placed in T cell enrichment nylon wool columns and maintained for 45 min in a humidified atmosphere with 5% CO_2_ at 37°C. The purity of the isolated cells was checked by flow cytometry analysis with PE-Cy5.5-conjugated anti-CD3 and FITC-conjugated anti-B220 staining (about 80% of T lymphocytes and 20% non-T cells, but mainly B220^+^ cells) and viability was checked using the 0.25% trypan blue dye exclusion method and found to be greater than 95%.

An in vitro study to show the direct effect of losartan or captopril in different splenic T cell responses was also performed. Aliquots containing 1.5×10^6^ cells from naive or vehicle-treated mice infected with *P. berghei* ANKA were distributed into 48-well plates (Corning Inc., New York, NY, USA), and treated or not for 30 min with 10^–6^ M losartan or 10^–6^ M captopril and maintained in a humidified atmosphere with 5% CO_2_ at 37°C prior to analysis.

### Transwell Migration Assay

Splenic T cells from naive mice or mice infected with *P. berghei* ANKA or T cells from vehicle-treated mice infected with *P. berghei* ANKA after culture treatment with 10^–6^ M losatan or 10^–6^ M captopril were resuspended at 5×10^5^ cells in 100 µl of RPMI 1640 supplemented with 1% BSA (assay medium). Cells were added to the upper chamber of 5.0-µm pore diameter Transwell tissue culture inserts (Millipore, Bedford, MA, USA), coated with fibronectin (10 µg/ml), laminin (10 µg/ml) or BSA (10 µg/ml), which was used as a negative control to discount unspecific migration. The inserts were placed in individual wells of a 24-well cell culture plate containing 600 µl of assay medium in the absence or presence of 10^−8^ M Ang II. Plates were incubated for 3 h at 37°C in 5% CO_2_. Transmigrated cells were collected from the lower chamber, counted, stained with fluorescent antibodies against CD3, CD4 and CD8 and analyzed by flow cytometry. The results are expressed as the number of transmigrated cells through endothelial basal membrane proteins minus the number of transmigrated cells through BSA×10^4^.

### Cell Adhesion Assay

The vascular endothelial cell line, tEnd.1, was cultured in RPMI 1640 medium supplemented with 10% heat-inactivated FBS, 2 mM l-glutamine, 100 IU/ml penicillin, and 100 µg/ml streptomycin. The cells were plated onto 24-well culture plates (Nunc, Rochester, NY, USA) and incubated (10^4^ cells/well) at 37°C in a humidified incubator containing 5% CO_2_ chambers for 24 h. Before each experiment, tEnd.1 cells were treated for 18 h with recombinant mouse TNF-α (10 ng/ml). Splenic T cells recovered from naive mice or mice infected with *P. berghei* ANKA or T cells from vehicle-treated mice infected with *P. berghei* ANKA pretreated with 10^–6^ M losatan or 10^–6^ M captopril were resuspended at 1×10^6^ cells in 100 µl of RPMI 1640 supplemented with 10% FBS (assay medium). The pretreated T cells were then allowed to adhere to tEnd.1 cultures for 1 h [21]. After incubation, non-adhered cells were washed out with phosphate-buffered saline (PBS) and the remaining cells were analyzed under an optical microscope after Diff-Quick staining. The percentage of adherent cells was described as the number of tEnd.1 cells containing T lymphocytes in relation to the total number of tEnd.1 cells in each well, after analysis of at least 10 random microscopic fields.

### Filamentous Actin Staining

Splenic T cells from naive mice or mice infected with *P. berghei* ANKA or T cells from vehicle-treated mice infected with *P. berghei* ANKA after culture treatment with 10^–6^ M losatan or 10^–6^ M captopril were resuspended at 5×10^5^ cells in 100 µl of RPMI 1640 supplemented with 1% BSA (assay medium). T cells (5×10^5^) were allowed to adhere for 1 h to coverslips treated previously with fibronectin (10 µg/ml) and fixed at room temperature with 4% paraformaldehyde (v/v) in PBS, pH 7.0. Thereafter, cells were permeabilized with 3% NP-40 for 40 min, followed by 15 min in acetone at –20°C. T cells were quenched using 50 mM ammonium chloride solution and 3% BSA in PBS for the next 20 min. Cells were covered with 0.4 units of Alexa Fluor 488-phalloidin (Invitrogen, Eugene, OR, USA) in methanol and incubated in a humidified chamber (1 h, 4°C). Cells were quenched in 3% BSA/PBS for 20 min and mounted in Vectashield mounting medium (Vector Laboratories, Burlingame, CA, USA). Cells were examined with a laser-scanning confocal microscope (Fluoview FV300, Olympus, Japan) under an oil immersion objective (60×). Images were obtained and processed using Fluoview 3.3 software (Olympus).

### Flow Cytometry Analysis

Splenic T cells isolated from mice were incubated with the appropriate concentration of anti-CD3 mAb, anti-CD4 mAb, anti-CD8 mAb, anti-CD25 mAb, anti-CD69 mAb, anti-CD62L mAb, anti-CD44 mAb, anti-perforin, anti-CD11a, anti-CCR2 mAb, anti-CCR5 mAb or IgG isotype controls for 30 min at 4°C, after incubation with anti-CD16/CD32 (Fc block) to block nonspecific binding sites. Analysis of surface markers was performed using the Summitt software after acquisition in a FACSCalibur flow cytometer (Becton Dickinson, San Jose, CA, USA). At least 10^4^ lymphocytes were acquired per sample. All data were collected and displayed on a log scale of increasing fluorescence intensity and presented as dot plots. Percentages of T lymphocytes were determined in a specific CD3^+^ T lymphocyte gate.

For intracellular and cytokine staining experiments, splenocytes were incubated with FITC-anti-CD4, PE-Cy5.5-anti-CD3 or APC-anti-CD25 for 1 h. After washing, cells were fixed and permeabilized with Citofix/Citoperm (BD Pharmingen, San Diego, CA, USA), and incubated with PE-anti-Foxp3 or PE-anti-IFN-γ or PE-anti-IL-4 or PE-anti-IL-17 or PE-anti-IL-10 or an isotype-matched antibody. The cells were then washed twice, resuspended in PBS and analyzed in a FACSCalibur cytofluorometer (BD Biosciences, Franklin Lakes, NJ, USA). Viable cells were gated by forward and side scatter.

### IC_50_ Value Determination for Losartan and Captopril in the Percentage of CD69^+^ T Cells

The IC_50_ values for Losartan and Captopril in the prevention of T cell activation were determined by treating *Plasmodium berghei ANKA* infected-mice with different doses of each drug, daily, for 6 days (0.01–200 mg/kg/d). The IC_50_ values were calculated by nonlinear regression analysis with best-fit of the experimental values using GraphPad Prism program (GraphPad, San Diego, CA, USA), assuming that the dose-response curve has a standard slope, equal to a Hill slope of 1. The results are expressed in relation to the maximal percentage of CD69^+^ T cells observed in mice infected with *P. berghei* ANKA treated with vehicle (100%). Experiments were performed in triplicate.

### Immunodetection of Angiotensin Receptors

Splenic T lymphocytes from naive mice or vehicle-, losartan- and captopril-treated mice infected with *P. berghei* ANKA were resuspended and lysed on ice for 40 min in Ripa buffer (25 mM Tris–HCl, pH 7.5, 150 mM NaCl, 1 mM EDTA, 1% Triton X-100, 0.5% deoxycholate and 0.1% sodium dodecyl sulfate) freshly supplemented with phosphatase and protease inhibitors (10 mM NaF, 5 mM Na_3_VO_4_, 5 mM Na_4_P_2_O_7_ and 1× protease inhibitor cocktail; Roche, Indianapolis, IN, USA). The final protein concentration in each condition was determined by the Bradford method [22] using BSA as a standard. Aliquots containing 30 µg of protein were resuspended in sodium dodecyl sulfate (SDS)-polyacrylamide gel electrophoresis loading buffer, resolved on sodium dodecyl sulfate 9% acrylamide gels and transferred onto Immobilon-P membranes (Millipore, Bedford, MA, USA). After blocking with 5% nonfat dry milk/Tris-buffered saline containing 0.1% Tween 20 for 1 h at room temperature, membranes were probed overnight at 4°C with primary specific antibodies, followed by horseradish peroxidase-labeled secondary antibodies (Amersham Biosciences, Piscataway, NJ, USA) and visualized with ECL®-plus reagent (Enhanced Chemiluminescence, Amersham Biosciences). Mouse monoclonal anti-human AT_1_ and rabbit polyclonal anti-human AT_2_ were obtained from Santa Cruz Biotechnology, Inc. (Santa Cruz, CA, USA). The probed membranes were stripped with Re-Blot Plus Western blot stripping solution (Millipore) for 30 min at room temperature and reprobed with rabbit polyclonal β-actin to detect total levels of protein.

### Enzyme-Linked Immunoabsorbent Assay

Levels of TNF-α, IFN-γ and IL-10 in the serum collected from naive or vehicle-, losartan- and captopril-treated mice infected with *P. berghei* ANKA were evaluated by sandwich ELISA using matched antibody pairs from R&D Systems (Minneapolis, MN, USA), according to the manufacturer’s instructions. Results are expressed as pg/ml.

### Evaluation of Blood-brain Barrier Disruption

Mice received an intravenous (i.v.) injection of 1% Evans blue (Sigma-Aldrich, São Paulo, Brazil). One hour later, mice were euthanized and their brains were weighed and placed in formamide (2 mL, 37^o^ C, 48 h) to extract the Evans blue dye from the brain tissue. Absorbance was measured at 620 nm (spectramax 190, Molecular Devices, CA, USA). The concentration of Evans blue was calculated using a standard curve. The data are expressed as µg of Evans blue per g of brain tissue.

### Peripheral Blood Mononuclear Cell Labeling and Adoptive Transfer

Peripheral blood cells were submitted to Histopaque-1119 and Histopaque-1077 (Sigma-Aldrich) double density gradient centrifugation methods to recover peripheral blood mononuclear cells. Cells were washed with sterile PBS and incubated with a 1 µM solution of CFSE (Invitrogen) for 30 min at 4°C. Cells were then washed, resuspended in sterile saline, and adoptively transferred into injected mice by i.v. injection (10^7^ cells/mouse) [23]. One day after adoptive transfer, recipient mice were infected and treated as described above (vehicle-, losartan- or captopril-treated infected mice). Five days after infection, the CFSE-labeled T lymphocytes adhering to the brain microvasculature were evaluated as follows. Mice were perfused with PBS and the brains were removed, homogenized and incubated with collagenase IV (0.05%, 30 min, 37°C; Invitrogen). The cell suspensions were washed, the pellet resuspended in 40% Percoll solution (Percoll Plus; GE Healthcare) and layered on an 80% Percoll solution. After centrifugation, cells at the interphase between both Percoll layers were collected. Subsequently, leukocytes from the brain were washed, counted with a hemacytometer, and evaluated by flow cytometry (FACScalibur). CD8^+^CD69^+^ and CFSE-labeled T cells were set in the T-lymphocyte region determined in an FSC and SSC dot plot and confirmed by positive CD3– fluorescent staining. Additionally, in another experimental group, 6 days post-infection, at time infected mice have cerebral malaria signs, leukocytes were recovered from the brain of mice from the different groups as described above, counted and submitted to FACS analysis to determine the number of sequestered CD3^+^CD8^+^ T cells.

### Statistical Analysis

All experiments were carried out with at least 4 or 5 animals per experimental group (non-infected, vehicle-, losartan- or captopril-treated infected mice). Each animal was considered individually in each determination and the data are expressed as the mean ± SD. The data were analysed by one-way analysis of variance (ANOVA), considering the treatments as factors. The significance of the differences was verified by the Bonferroni post test. Statistical analysis was performed using absolute values. *P*<0.05 was considered statistically significant. In order to compare the percentage survival, the log-rank (Mantel–Cox) test was used and the significance level was set at *P*<0.05.

## Results

### Ang II Acts as a Co-stimulus for T Cell Activation and Differentiation during *P. berghei* ANKA Infection

In the first experimental group, we evaluated the possible involvement of Ang II in the early stage response of T lymphocytes during *P. berghei* ANKA infection. The percentage of spleen-derived CD4^+^ and CD8^+^ T cells expressing CD69 in noninfected (naive) mice and in mice infected with *P. berghei* ANKA treated or not with RAS inhibitors (losartan or captopril) was examined. Treatment with losartan or captopril was started immediately after infection, in a dose at least 10 or 20 times above the IC_50_ for Captopril (IC_50_ = 1.940 mg/Kg/d) or Losartan (IC_50_ = 0.966 mg/Kg/d), respectively in relation to CD69 expression (Fig. S1).

**Figure 1:**
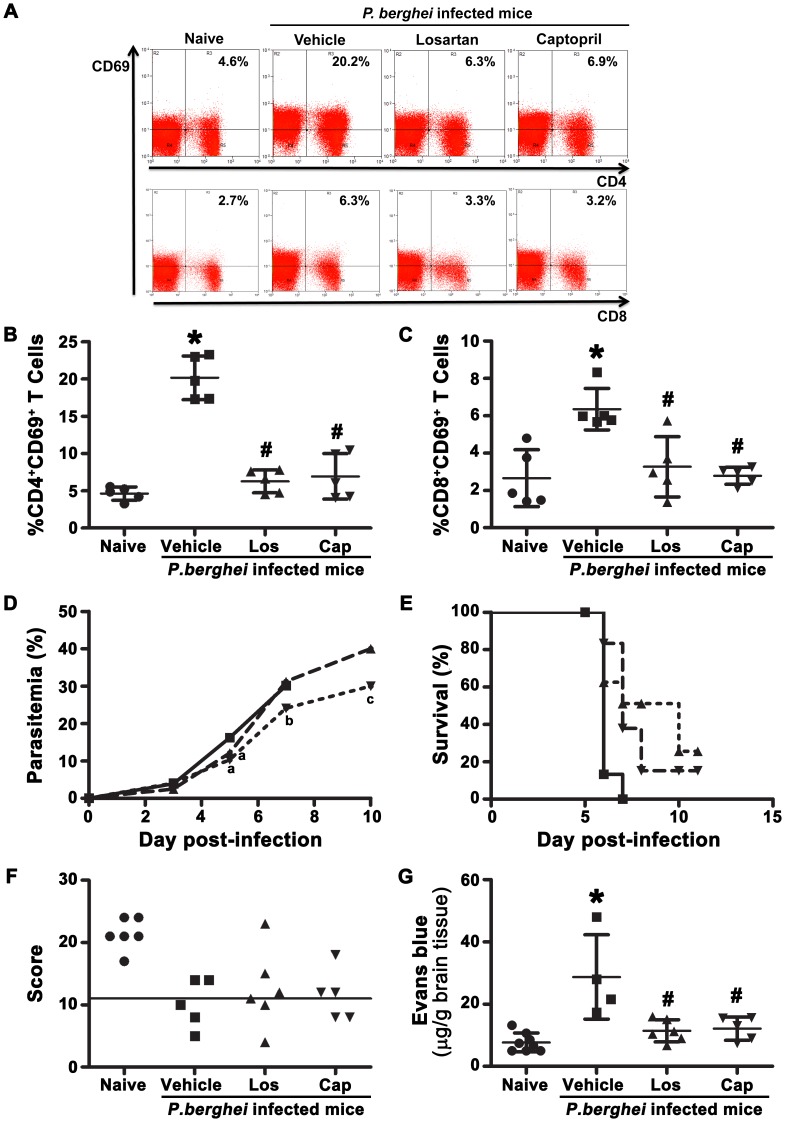
Losartan or captopril treatment inhibited *P.berghei* ANKA-induced splenic T cell activation. C57BL/6 mice were infected with *P. berghei* ANKA and treated with vehicle, losartan or captopril by gavage. T cells were isolated at day 6 post infection, stained with fluorescent antibodies, and analyzed by flow cytometry. (A) Representative dot plots of CD4^+^CD69^+^ and CD8^+^CD69^+^ T cells obtained from gated CD3^+^ cells. Percentage of CD4^+^CD69^+^ T cells (B) and CD8^+^CD69^+^ T cells (C). Parasitemia was determined by Giemsa-stained blood smears in vehicle- (solid line), losartan- (dotted line) or captopril- (dash line) treated mice (D). Mortality was checked daily in vehicle- (▪), losartan- (▴) or captopril- (▾) treated mice (E). Behavioral analysis was assessed as described in Materials and Methods (F). Evans blue dye extraction from the brain tissue to evaluate blood-brain barrier disruption (G). The results are expressed as means±SD. Statistically significant compared with values for *naive mice (*p*<0.05) and #vehicle-treated mice infected with *P. berghei* ANKA (*p*<0.05). Statistically significant compared with values for vehicle-treated infected mice at ^a^day 5, ^b^day 7 or day ^c^10 post-infection.

We observed that the percentage of CD4^+^CD69^+^ (Fig. 1A,B) and CD8^+^CD69^+^ (Fig. 1A,C) T cells was 4- or 3-fold higher, respectively, in response to malaria infection, similar to the results observed by Elias et al [24]. The treatment with 20 mg/kg losartan, an AT_1_ receptor antagonist, and 20 mg/kg captopril, a well-known angiotensin-converting enzyme (ACE) blocker, avoided this effect, maintaining CD4^+^CD69^+^ and CD8^+^CD69^+^ T cells at the naive level (Fig. 1B,C). The same profile was observed when the absolute number of cells was analyzed (Fig. S2A,B). Based on the mean intensity of fluorescence (MIF) analysis, CD69 expression increased 58% during infection in both T cell subsets, indicating that the activation process was present and the treatment with drugs impaired it (Fig. S2C,D). We also analyzed T cells expressing CD25 (excluding CD25^+^Foxp3^+^ T cells), another activation marker of T cells. We observed that *P. berghei* ANKA infection induced a strong increase in the percentage of CD25^+^ T cells in relation to naive cells. The treatment of mice infected with *P. berghei* ANKA with losartan or captopril partially reduced the generation of CD25^+^ T cells in the spleen of infected mice (data not shown). Since treatments impaired T cell activation, in the next step, we evaluated the impact of drugs on the outcome of infection. We observed reduced blood parasitemia in treated mice up to 5 days post infection; however, at day 7, the parasitemia level in losartan-treated mice was significantly lower than in the control group but it suddenly increased to 30% on day 10 (Fig. 1D). Captopril-treated animals achieved 40% parasitemia on day 10. A considerable survival benefit was observed in losartan- or captopril-treated mice (Fig 1E). CM neurological signs determined based on a scoring system [20] achieved a score value of 11.6 with both treatments, which is not considered positive to cerebral malaria (Fig. 1F). Moreover, cerebral edema clearly detected in *P. berghei* ANKA infected mice was significantly inhibited with both losartan and captopril treatment (Fig. 1G). Although we observed survival benefit and reduction in cerebral edema in the group of animals that received losartan or captopril, the SHIRPA score was only slightly improved. These data suggest partial protection from cerebral malaria.

Based on these observations, in the next experimental group, we evaluated the effect of Ang II on T cell sequestration to the brain tissue and local activation (Fig. S3). The frequency of CFSE-labeled T lymphocytes was evaluated in the brain of mice in the different experimental groups after perfusion. The results revealed a strong reduction in the percentage of CFSE^+^ T cells in the brain of captopril-treated infected mice (Fig. S3A). Additionally, at the time mice have cerebral malaria signs in the control group (day 6 post-infection), the number of CD8^+^ T cells sequestered in the brain reduced considerably after captopril treatment (Fig. S3B). Although there was no significant decrease in the frequency of this population in the losartan-treated group, the percentage of CD8^+^CD69^+^ T cells dropped considerably (Fig. S3B). These results suggest that Ang II could play an important role in driving homing, sequestration or activation of T lymphocytes in the brain of infected animals.

We also evaluated the effect of Ang II in the course of primary effector response by following the changes in CD44 and CD62L expression levels in spleen-derived T cells. Naive CD4^+^ and CD8^+^ T cells, characterized as CD62L^high^CD44^low^, were reduced by 50% after *P. berghei* ANKA infection. However, this reduction was attenuated when infected mice were treated with losartan or captopril (Fig. S4A–C). CD62L^high^CD44^high^ and CD62L^low^CD44^high^ T cell populations induced by infection were both reduced with treatment (Fig. S4A,D–G). The CD62L^low^CD44^high^ profile was induced differently in both CD4^+^ and CD8^+^ T cell subsets by *P. berghei* ANKA infection. CD4^+^ T cells increased 54%; an increase of 283% was observed in the CD8^+^ T cell subset (Fig. S4A,F,G).

**Figure 2:**
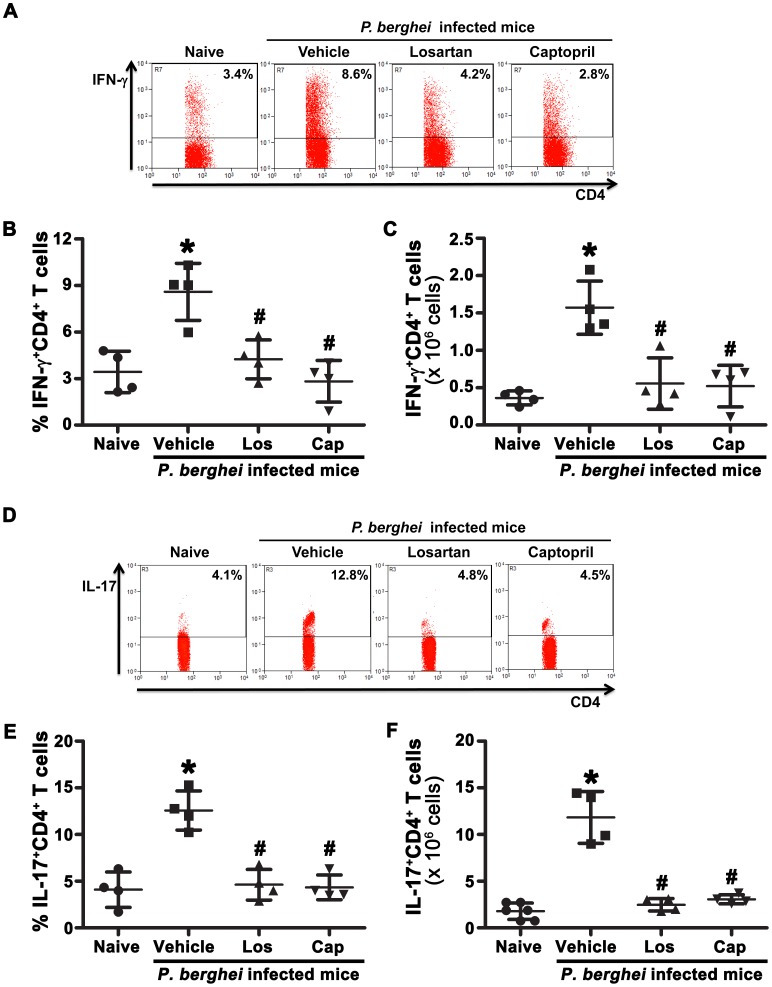
Lack of Ang II production or AT_1_ blockade impaired IFN-γ and IL-17 cytokine production by splenic CD4^+^ T cells during *P.berghei* ANKA infection. T cells from naive mice and mice infected with *P. berghei* ANKA treated with vehicle, losartan or captopril by gavage were isolated at day 6 post infection, stained with fluorescent antibodies and analyzed by flow cytometry. Representative dot plots of IFN-γ^+^CD4^+^ (A) or IL-17^+^CD4^+^ (D) T cells obtained from gated CD3^+^ cells. The percentage (B) and absolute number of IFN-γ^+^CD4^+^ (C) and the percentage (E) and absolute number of IL-17^+^CD4^+^ (F) T cells were determined. The results are expressed as means±SD. Statistically significant compared with values for *naive mice (*p*<0.05) and #vehicle-treated mice infected with *P. berghei* ANKA (*p*<0.05).

Thus, our results show that Ang II through the AT_1_ receptor is involved in the activation of CD4^+^ and CD8^+^ T cells during *P. berghei* ANKA infection. We then verified whether Ang II affects the functional properties of the CD4^+^ T cell population by evaluating the cytokine profile produced by these cells. The frequency and absolute number of spleen-derived IFN-γ, IL-4 and IL-17-producing CD4^+^ T cells in the different experimental groups was determined (Fig. 2). *P. berghei* ANKA infected mice showed an increase in IFN-γ and IL-17-producing CD4^+^ T cells that was completely abolished with losartan or captopril treatment (Fig. 2A–F). On the other hand, none of the treatments seemed to modulate IL-4 production, which was barely detected in both noninfected (naive) or infected mice (data not shown).

**Figure 3:**
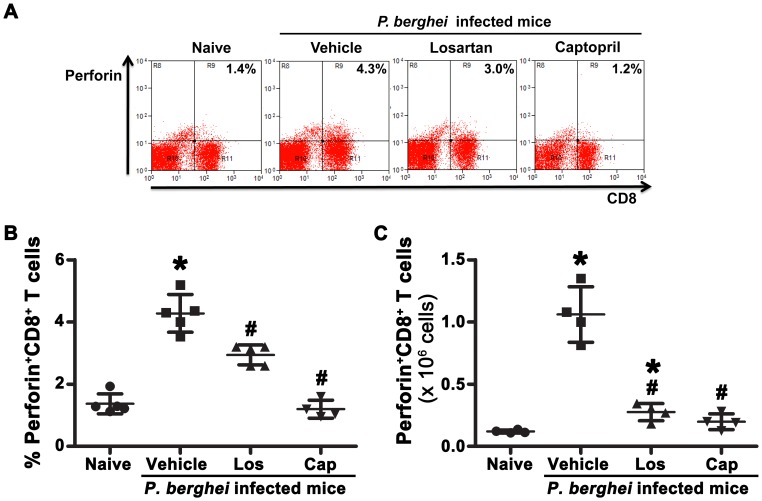
Ang II influences the production of perforin by splenic CD8^+^ T cellsduring *P. berghei* ANKA infection. T cells from naive mice and mice infected with *P. berghei* ANKA treated with vehicle, losartan or captopril by gavage were isolated at day 6 post infection, stained with fluorescent antibodies and analyzed by flow cytometry. (A) Representative dot plots of perforin^+^CD8^+^ T cells obtained from gated CD3^+^ cells. The percentage (B) and absolute number (**C**) of perforin^+^CD8^+^ T cells were determined. The results are expressed as means±SD. Statistically significant compared with values for *naive mice (*p*<0.05) and #vehicle-treated mice infected with *P. berghei* ANKA (*p*<0.05).

In relation to perforin-expressing CD8^+^ T cells, a marker of cytolytic capacity, we observed a 4-fold increase in the frequency and 10-fold increase in the absolute number of these cells in the spleen of mice infected with *P. berghei* ANKA treated with vehicle. Losartan treatment in the mice infected with *P. berghei* ANKA partially reduced the percentage of CD8^+^perforin^+^ T cells in the spleen; the treatment with captopril completely inhibited the expression of perforin in splenic CD8^+^ T cells (Fig. 3A–C). These results indicate that Ang II is somehow involved in a cytolytic lymphocyte function during *P. berghei* ANKA infection.

**Figure 4:**
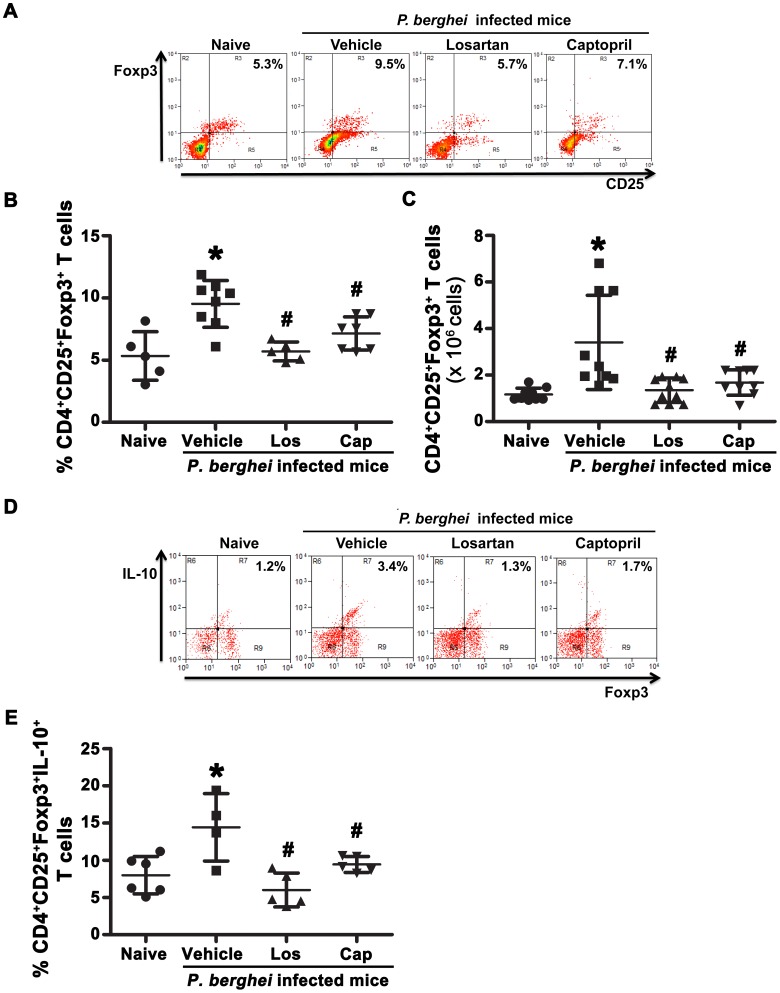
Influence of Ang II in the expansion of inducible Foxp3^+^ regulatory T cells and IL-10 production during *P.*berghei ANKA infection. C57BL/6 mice were infected with *P. berghei* ANKA and treated with vehicle, losartan or captopril by gavage. T cells were isolated at day 6 post infection, stained with fluorescent antibodies and analyzed by flow cytometry. Representative dot plots of CD25^+^Foxp3^+^ T cells obtained from gated CD4^+^CD3^+^ cells (A) and IL-10^+^Foxp3^+^ T cells obtained from gated CD4^+^CD25^+^ T cells (D). The percentage (B) and absolute number of CD4^+^CD25^+^Foxp3^+^ T cells (C) and the percentage of IL-10^+^CD4^+^CD25^+^Foxp3^+^ T cells (E). The results are expressed as means±SD. Statistically significant compared with values for *naive mice (*p*<0.05) and #vehicle-treated mice infected with *P. berghei* ANKA (*p*<0.05).

It is already known that *Plasmodium* infection is able to induce a regulatory response in both human and murine infections as a mechanism to inhibit both protective and overwhelming inflammatory responses induced by malaria [25]–[31]. Here, we have demonstrated that Ang II is involved in the expansion of CD4^+^CD25^+^Foxp3^+^ T cells (Treg) because losartan or captopril treatment abolished the *Plasmodium*-induced increase in this population, represented by a 2-fold increase in the frequency and number of these cells (Fig. 4A–C). Moreover, IL-10–producing Tregs were reduced to naïve mice levels by losartan and captopril (Fig. 4D,E). These results demonstrate that Ang II has an important role in driving the induction of splenic effector IFN-γ-, IL-17- or IL-10-producing CD4^+^ T cells, Treg phenotype and in the effector capacity of CD8^+^ T cells, concerning perforin expression during *P. berghei* ANKA infection.

### Ang II is an Important Endogenous Mediator for the Adhesion/transmigration of Malaria-activated T Lymphocytes

**Figure 5:**
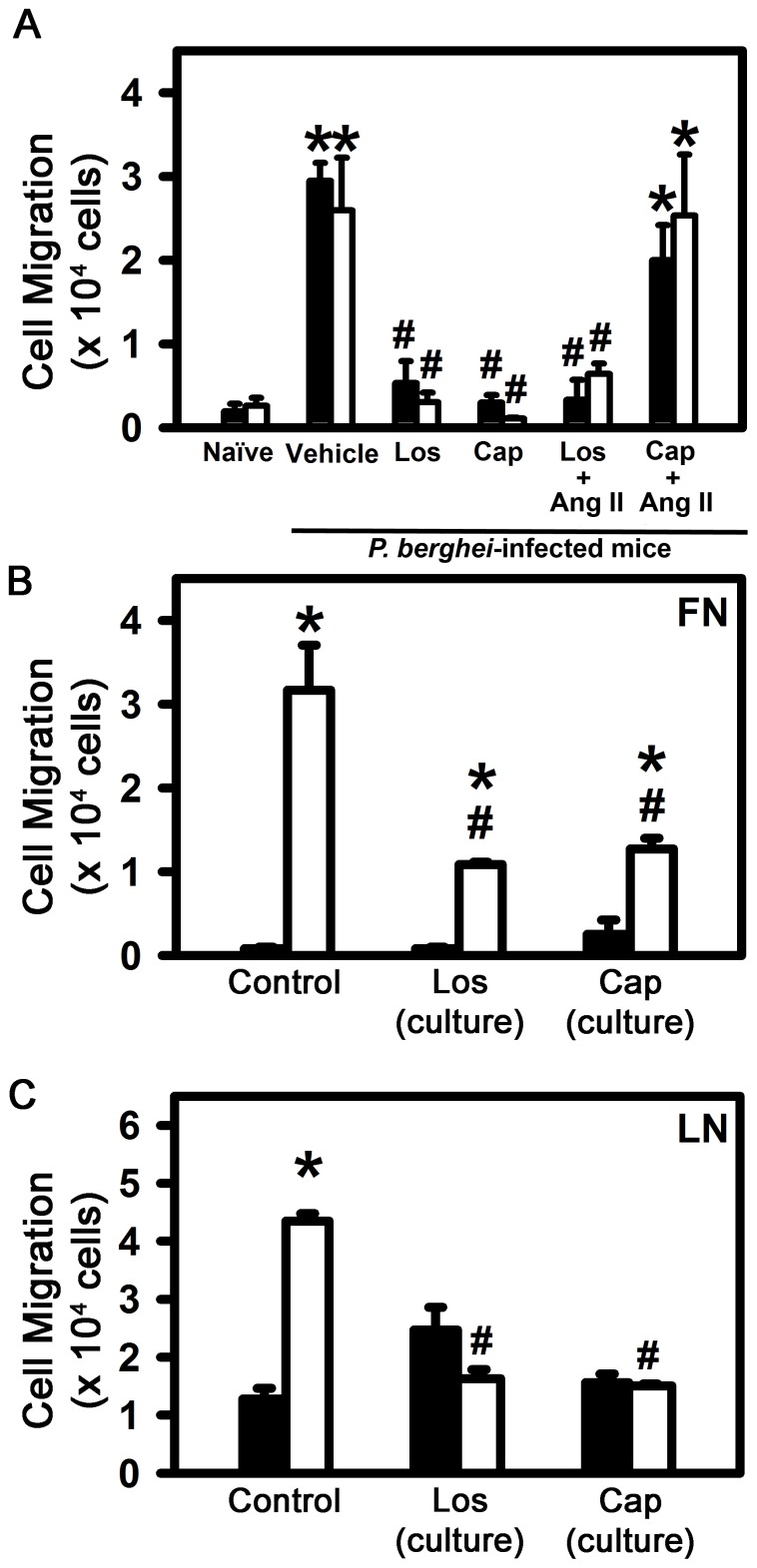
Endogenous Ang II induces transmigration of T lymphocytes during *P.berghei* ANKA infection on contact with endothelial basal membrane proteins. 5×10^5^ splenic T lymphocytes isolated from naive mice or mice infected with *P. berghei* ANKA treated with vehicle, losartan or captopril by gavage were added to the upper chamber of Transwell culture inserts (5.0 µm pore diameter) previously coated with fibronectin (10 µg/ml), laminin (10 µg/ml) or BSA (10 ug/ml) as negative control. The inserts were placed in individual wells of a 24-well cell culture plate containing 600 µl of assay medium in the absence or presence of 10^−8^ M Ang II as depicted in each panel and incubated for 3 h at 37°C in 5% CO_2_. (A) Transmigrated cells through the fibronectin matrix (black bars) and through the laminin matrix (open bars), minus the number of cells that migrated from BSA×10^4^. The results are expressed as means±SE. Statistically significant compared with values for *naive mice (*p*<0.05) and #vehicle-treated mice infected with *P. berghei* ANKA (*p*<0.05). Transmigrated T lymphocytes from naive (black bars) or vehicle-treated mice infected with *P. berghei* ANKA (open bars) that received treatment in culture with 10^–6^ M losartan or 10^–6^ M captopril for 30 min before the transmigration assay, through the fibronectin matrix (B) and through the laminin matrix (C) minus the number of cells that migrated from BSA×10^4^. The results are expressed as means±SE. Statistically significant compared with values for *naive mice in the control condition (*p*<0.05) and #vehicle-treated mice infected with *P. berghei* ANKA in the control condition (*p*<0.05).

In the next group of experiments, we assessed the influence of endogenous Ang II in the control of T cell traffic during malaria infection. To measure T cell transmigration, we used a Transwell system with the insert covered with fibronectin or laminin, two different endothelial basal membrane proteins. First, we evaluated whether blocking the endogenous production of Ang II by captopril or the impairment of AT_1_-mediated Ang II signaling by losartan interfered in the transmigration capacity of spleen-derived T lymphocytes from mice infected with *P. berghei* ANKA. Regardless of the matrix used, cells from infected mice readily migrated compared with naive cells (Fig. 5A). The treatment of infected mice with losartan or captopril reduced the intrinsic transmigration capacity of these cells more than 80%. The addition of 10^–8^ M Ang II to the lower chamber did not restore the migration of T cells obtained from mice infected with *P. berghei* ANKA treated with losartan. Nevertheless, T lymphocytes from infected mice treated with captopril promptly increased the transmigration in response to exogenous Ang II at levels similar to vehicle-treated infected mice (Fig. 5A). These data confirm the role of AT_1_ on the effect of Ang II in T cell transmigration. However, it is not clear whether Ang II acts directly on T cells or its effect is mediated by non-T cells such as endothelial or dendritic cells [32], [33].

To address this question, we isolated T cells from noninfected or infected mice and treated them *in vitro* with 10^–6^ M losartan or 10^–6^ M captopril. Fig. 5B and C show that treatment of the isolated cells (*in vitro*) with these drugs also reduced the number of transmigrating cells derived from infected mice in both matrixes. The same phenomenon was not observed in naive cells obtained from non-infected mice. When cells derived from infected mice were exposed to exogenous Ang II (10^–8^ M), we did not observe any modification in the migration profile of the cells (data not shown), corroborating the idea that activated T cells are able to produce endogenous Ang II at levels sufficient to induce migration when in contact with fibronectin or laminin matrixes, as observed previously [17], [34].

**Figure 6:**
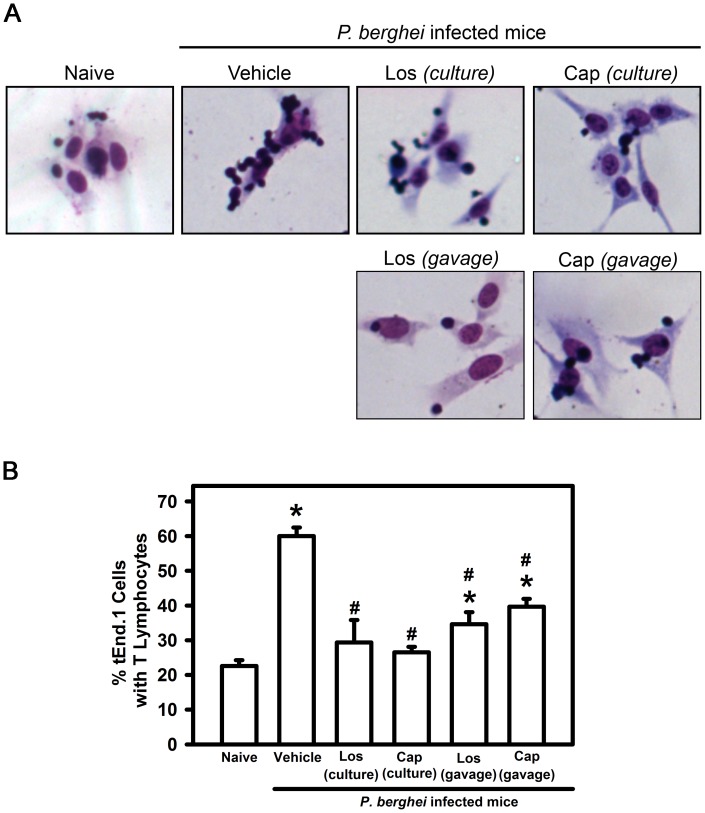
Effect of Ang II on adhesion of T lymphocytes to murine-activated endothelial cells (tEnd.1 cells) during *P.berghei* ANKA infection. T lymphocytes from naive mice and mice infected with *P. berghei* ANKA treated with vehicle, losartan or captopril by gavage were isolated at day 6 post infection and co-cultured with TNF-α-activated tEnd.1 cells for 1 h. In addition, T lymphocytes from vehicle-treated mice infected with *P. berghei* ANKA were treated in culture with 10^–6^ M losartan or 10^–6^ M captopril for 30 min prior to co-incubation with TNF-α-activated tEnd.1 cells. (A) Representative microscope images showing endothelial cells containing T cells adhered after Diff-Quick staining. (B) The percentage of adherent cells described as the number of tEnd.1 cells containing lymphocytes in relation to the total number of tEnd.1 cells under each condition, after analysis of at least 10 random microscopic fields. The results are expressed as means±SE. Statistically significant compared with values for *naive mice (*p*<0.05) and #vehicle-treated mice infected with *P. berghei* ANKA (*p*<0.05).

Before transmigrating into inflamed tissue, leukocytes adhere firmly to activated endothelial cells. This is an important process involved in the sequestration of cells in different organs during malaria infection [2], [15], [35]–[38]. Thus, we determined the role of Ang II in T lymphocyte–endothelial cell interaction using a well-established lineage of murine endothelial cells (tEnd.1 cells) activated *in vitro* with 10 µg/ml TNF-α. These cells were co-cultured with T lymphocytes obtained from naive mice or mice infected with *P. berghei* ANKA treated *in vivo* or in culture with losartan or captopril. It was observed that (Fig. 6A,B) the percentage of endothelial cells with T cells firmly adhered was enhanced 3 times when splenic T cells from infected mice were used. *In vivo* treatment with losartan or captopril reduced by 50% the adhesion property of activated T lymphocytes with tEnd.1 cells. Moreover, the treatment of splenic T cells from infected mice with these drugs in culture completely abolished the adhesion of these cells to tEnd.1 cells indicating again the direct effect of Ang II on T lymphocytes.

### Cellular Mechanisms Involved in the Effect of Ang II in Adhesion/transmigration of Malaria-activated T Lymphocytes

**Figure 7:**
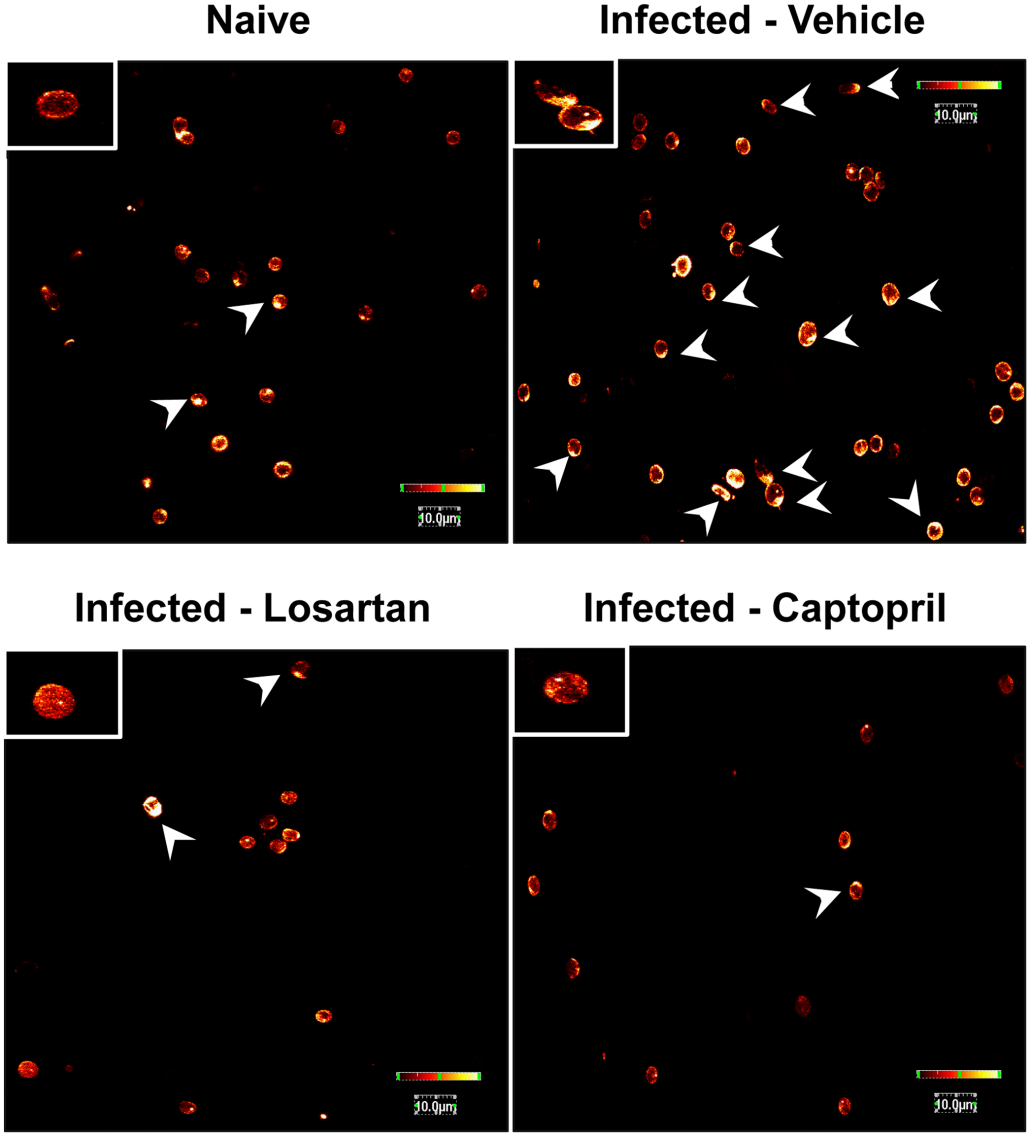
Ang II mediates F-actin polymerization in T lymphocytes during *P.berghei* ANKA infection on contact with endothelial basal membrane proteins. Splenic T lymphocytes isolated from naive mice and mice infected with *P. berghei* ANKA treated with vehicle, losartan or captopril by gavage were stimulated for 1 h with fibronectin followed by F-actin cytoskeleton staining by phalloidin-Alexa Fluor 488. Cells were examined by confocal microscopy. Arrows indicate cells presenting spread morphology. The color bar on the right of the images represents the relative scale of fluorescence intensity.

We investigated whether Ang II signaling could elicit changes to the cytoskeleton in T cells during *P. berghei* ANKA infection. Purified resting splenic T lymphocytes presented a spherical morphology and did not exhibit stress fibers, as shown by confocal microscopy analysis using Alexa 488 phalloidin staining (Fig. 7A, upper left panel). Splenic T cells isolated from vehicle-treated mice infected with *P. berghei* ANKA stimulated by interaction with the fibronectin matrix, exhibit intense staining of cortical F-actin fibers, indicating rearrangement of the actin cytoskeleton (Fig. 7B, upper right panel). T cells from mice infected with *P. berghei* ANKA treated *in vivo* with losartan or captopril showed a similar profile to the naive T cells, with diminished staining of F-actin fibers and just a few cells with spread morphology (Fig. 7C, lower panels).

**Figure 8:**
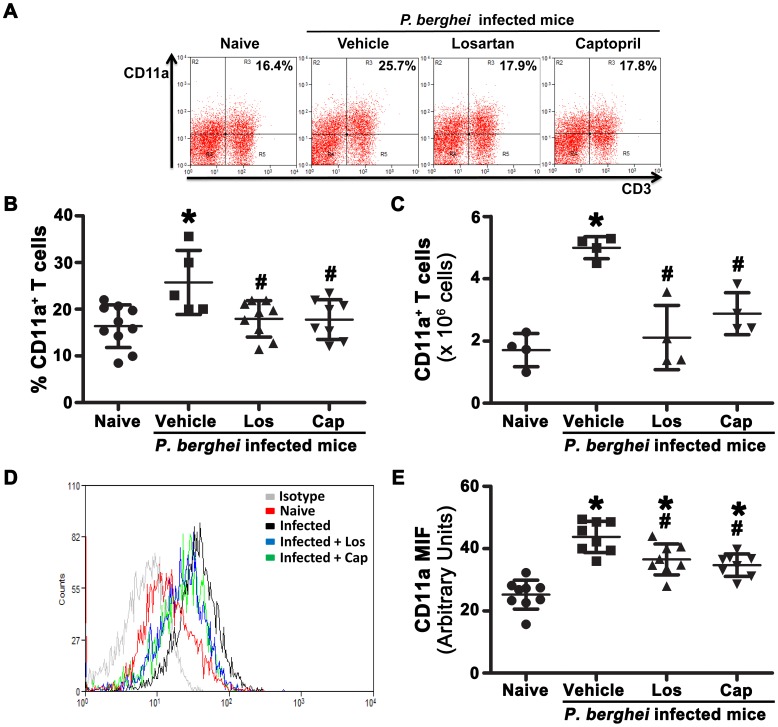
Ang II induces the upregulation of CD11a expression in splenic T cells during *P.berghei* ANKA infection. C57BL/6 mice were infected with *P. berghei* ANKA and treated with vehicle, losartan or captopril by gavage. T cells were isolated at day 6 post infection, stained with fluorescent antibodies and analyzed by flow cytometry. (A) Representative dot plots of CD3^+^CD11a^+^ T cells. The percentage of CD11a^+^ T cells (B) and absolute number of CD11a^+^ T cells (C) were calculated. CD11a expression was analyzed by MIF on gated CD3^+^ T cells (D, E). The results are expressed as means±SD. Statistically significant compared with values for *naive mice (*p*<0.05) and #vehicle-treated mice infected with *P. berghei* ANKA (*p*<0.05).

**Figure 9:**
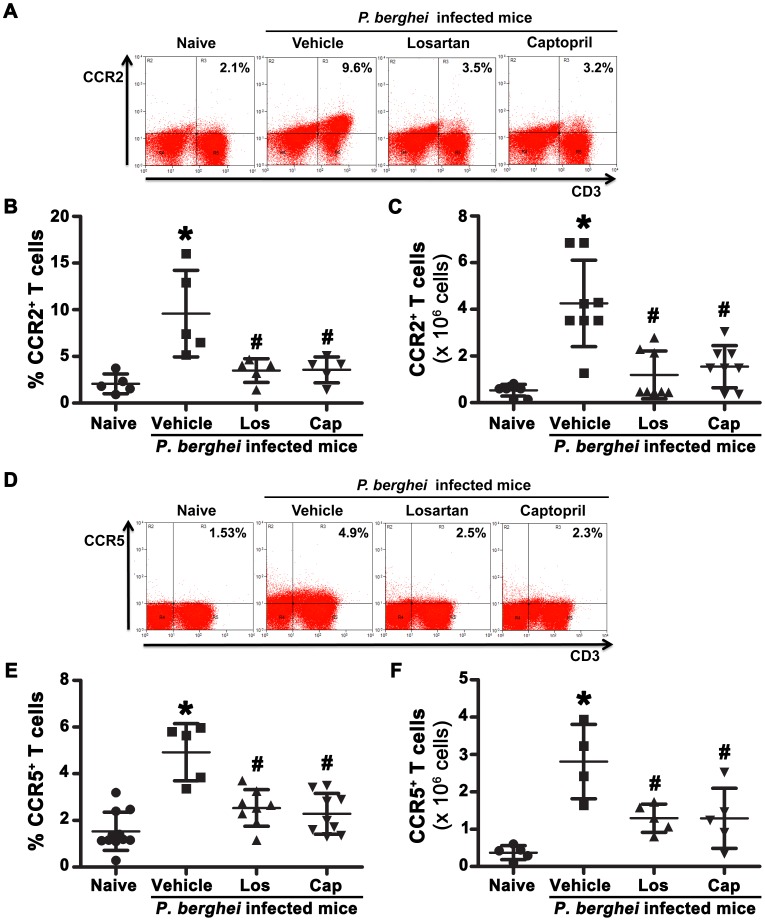
Losartan or captopril treatment reduces CCR2 expression in splenic T cells during *P.berghei* ANKA infection. C57BL/6 mice were infected with *P. berghei* ANKA and treated with vehicle, losartan or captopril by gavage. T cells were isolated at day 6 post infection, stained with fluorescent antibodies and analyzed by flow cytometry. Representative dot plots of CD3^+^CCR2^+^ (A) or CD3^+^CCR5^+^ (D) T cells. The percentage of CCR2^+^ T cells (B) or CCR5^+^ T cells (E), and the absolute number of CCR2^+^ T cells (C) or CCR5^+^ T cells (F) were calculated. The results are expressed as means±SD. Statistically significant compared with values for *naive mice (*p*<0.05) and #vehicle-treated mice infected with *P. berghei* ANKA (*p*<0.05).

Another mechanism by which Ang II could induce T lymphocyte adhesion/migration involves the control of adhesion molecules and/or chemokine receptor expression. The expression of CD11a (LFA-1), an adhesion molecule expressed in activated T cells during *P. berghei* ANKA infection, was analyzed by flow cytometry [39]. *P. berghei* ANKA infection upregulates CD11a expression in splenic T cells and treatment with losartan or captopril partially inhibited it (Fig. 8). FACS analysis of 2 important chemokine receptors known to promote T cell chemotaxis to inflamed tissues, such as CCR2 and CCR5, which are known to be involved in the development of severe malaria and CM, respectively, was performed [40], [41]. The percentage of CCR2^+^ or CCR5^+^ T cells during infection was increased in relation to naive mice (Fig. 9A–F); *in vivo* treatment with losartan or captopril abolished this effect. Based on MIF analysis, we suggest that both CCR2 and CCR5 are upregulated in T cells during *P. berghei* ANKA challenge; this was blocked by *in vivo* treatment with losartan or captopril (Fig. S5).

**Figure 10:**
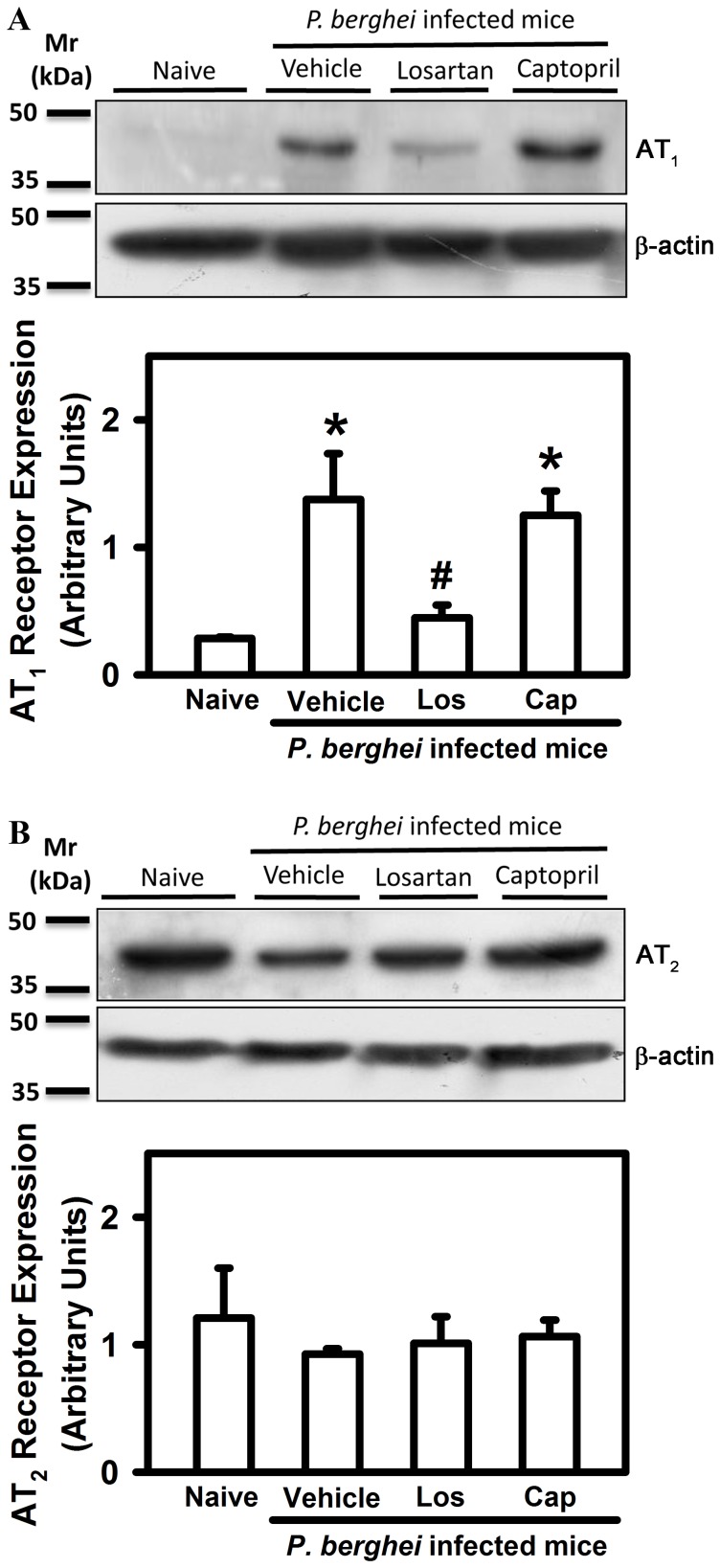
*P.berghei* ANKA infection induces upregulation of AT_1_ but not AT_2_ Ang II receptor expression in splenic T cells. 30 µg of T cell lysates from naive mice (lane 1), or mice infected with *P. berghei* ANKA treated with vehicle (lane 2), losartan (lane 3), or captopril (lane 4), by gavage, underwent immunoblotting detection of (A) AT_1_ or (B) AT_2_ Ang II receptors. AT_1_R or AT_2_R expression was normalized by β-actin expression. The results are expressed as means±SE. *Statistically significant compared with naive T cells (*p*<0.05).

In a previous paper, we observed that the expression of AT_1_ receptor in naive cells is low [17]. Here, we observed that AT_1_ mediated the effects of Ang II on the *P. berghei* ANKA infection. We decided to investigate whether AT_1_ receptor expression is modulated by *P. berghei* ANKA infection. *P. berghei* ANKA infection induced upregulation of AT_1_ receptor, but AT_2_ expression was not changed (Fig. 10). The increase in AT_1_ expression was blocked uniquely by losartan treatment, indicating a dependence of Ang II binding and signaling through AT_1_ receptor in inducing, by a positive feedback loop, its proper upregulation (Fig. 10A).

These results indicate that the cellular mechanism mediating the effect of Ang II during T cell immune response to *P. berghei* ANKA infection involves an increase in AT_1_ receptor, conformational changes in the actin cytoskeleton on contact with endothelial basal membrane proteins, and an increase in CD11a (LFA-1), CCR2 and CCR5 expression.

### Ang II Regulates the Plasma Concentration of Proinflammatory Cytokines during *P. berghei* ANKA Infection

In order to verify the influence of Ang II in the systemic modulation of proinflammatory cytokines, the plasma of naive or infected mice treated or not with losartan or captopril was used to determine the plasma concentration of TNF-α and IFN-γ. As depicted in Fig. S6A and B, there is a 5-fold and a 10-fold increase in the levels of TNF-α and IFN-γ, respectively, in the plasma of mice infected with *P. berghei* ANKA. The treatment of infected mice with losartan completely blocked malaria-induced production of TNF-α and partially suppressed the increase in IFN-γ in plasma. Captopril had no significant effect in the regulation of this proinflammatory cytokine production during malaria infection.

## Discussion

Immune responses are important to parasitemia control during malaria infection. However, it is generally accepted that the same immunological response contributes to malaria pathogenesis, with a local organ-specific inflammatory response initiated by the presence of parasite and sustained by infiltrating cells within the vasculature [2], [15], [36]–[39]. In this work, we describe an important new role for an AT_1_ receptor-mediated effect of Ang II in T cell activation, migration and adhesion during *P. berghei* ANKA infection. These results show an important role of RAS in malaria pathogenesis opening new perspectives to an appropriate and effective therapeutic intervention for malaria disease.

Ang II has been recognized to be involved in key events of the inflammatory process [42]–[44]. Ang II participates in the onset and progression of inflammation by upregulating the expression of adhesion molecules, such as P-selectin, VCAM-1 and ICAM-1, in endothelial cells and activating monocytes to adhere to them [45]–[48]. Moreover, Ang II-induced NF-κB activity and MCP-1 expression result in mononuclear cell accumulation [49]–[53]. Ang II can also induce rapid neutrophil infiltration *in vivo* by enhancing CXC chemokines, IL-8 and MIP-2 [54]. However, a direct correlation between the modulation of these processes and malaria infection has not yet been demonstrated. Why do we infer that Ang II is an important factor involved in the accumulation of T cells in inflamed vessels during *P. berghei* ANKA infection? In the present work, we observed that Ang II modulates different aspects of the T cell response during malaria due to *P. berghei* ANKA infection: (1) adhesion into endothelial basal membrane proteins or activated endothelial cells; (2) intrinsic transmigration capacity; (3) activation profile; (4) commitment to a specific phenotype; and (5) development of a memory response. We do believe this is only the beginning of the story. Further experiments are necessary to show whether the Ang II signal is somehow involved in severe malaria pathogenesis, especially CM. Here, we have shown that, besides survival benefits, different signals involved in the development of cerebral malaria and the death of *P. berghei* ANKA-infected mice were attenuated with treatments, such as cerebral edema as well as sequestration or activation of brain-derived T cells. Only a slight improvement in the behavioral analysis was observed, which could suggest that the effect of losartan and captopril is only partial in the protection from CM. Other factors not modulated by treatments are probably involved in the pathogenesis of the disease. However, mice in all groups still succumbed with high parasitemia levels. Our results and those in the literature in different models of disease indicate that Ang II plays a common role in different inflammatory processes with characteristics specific to *P. berghei* ANKA infection.

One important question arises regarding the source of Ang II. The blockage of adhesion and migration observed after *in vitro* treatment with losartan or captopril of spleen-derived T cells from infected mice revealed Ang II is endogenously produced by T cells. This idea is supported by the observation that the addition of exogenous Ang II induced migration even in the presence of captopril but migration did not change in the presence of losartan. We have recently demonstrated that endogenously produced Ang II by T cells activated with α-CD3 *in vitro* is important for cell adhesion/migration [17]. These results agree with previous observations that lymphocytes have their own functional RAS able to produce Ang II at levels sufficient to control activation, differentiation and adhesion/migration capacity [16], [17], [34], [55].

It has been shown that Ang II-induced sequestration of mononuclear cells at arterioles and venules is mediated by the generation of CC chemokines [49]–[53]. In addition, it has been observed that Ang II-induced infiltration of T cells into the blood vessels during hypertension involves increases in CD44 and CCR5 [55]. Sarfo et al [56] suggested that CCR3 and CCR5 and their ligand RANTES/CCL5 are upregulated in the brain of patients with CM. The role of CCR5 in CM has been confirmed in CCR5-deficient mice [40]. These animals are less susceptible to CM and have less CD8^+^ T cells accumulated in the brain vasculature. The correlation between Ang II and CCR5 upregulation induced by *Plasmodium* antigens was demonstrated by treating the infected mice with losartan or captopril. It was also observed that there is an increase in the number of CCR5^+^ cells and CCR5 expression level in the T cell population from the spleen of infected mice relative to naive mice. Furthermore, a similar effect of Ang II on CCR5^+^ T cells was obtained in spleen-derived T cells activated *in vitro* with anti-CD3 [34].

The treatments with losartan or captopril also abolished the increase in CCR2^+^ T cells in the spleen of infected mice. However, the significance of our findings in the pathogenesis of malaria is not yet clear, because it has been shown that CCR2 signaling does not have any influence on the development of CM in mice [41]. On the other hand, CCR2 expression is enhanced in the brain of infected mice and the upregulation of this chemokine receptor is important for the migration and accumulation of CD4^+^ T cells in the brain during CM [41]. Thus, it is plausible to imagine that Ang II-induced CCR2 expression in T cells is involved in the recruitment of CD4^+^ T cells to the brain during CM.

Molecular events triggered by TCR engagement or chemokine receptor activation represent the so-called inside-out events that induce conformational changes in LFA-1 molecules on the surface of T cells enhancing its affinity for ICAM-1 [57]. Following this idea, we showed that losartan or captopril treatment reduced the expression of CD11a (LFA-1) molecules. Furthermore, it was shown that these modifications are associated with a decrease in cell adhesion, cytoskeleton rearrangement, cell spreading and cell migration. Similarly, Crowley et al [58] showed that Ang II induces actin polymerization as a result of TCR engagement followed by integrin signaling in cultures of anti-CD3 activated T cells.

Besides adhesion/migration properties, Ang II has been also described to modulate T cells activation [16], [17], [34], [55]. Here we demonstrated that blockers of RAS significantly inhibited *in vivo* the expression and frequency of CD69^+^ T cells derived from infected mice. However, that Ang II could affect T cells directly or rely on the impairment of the adaptive response established by reducing dendritic cell function as described by Nahmod et al [32], [33] in mice lacking AT_1a_ receptor.

In addition, we have shown that Ang II could have a role in the differentiation and maintenance of central memory CD4^+^ or CD8^+^ T cells, because losartan or captopril reduced these populations. Memory capacity is a key element in T cell responses to provide a level of protection that can be recruited immediately (within hours) to combat an acute infection [59], [60]. However, the mechanisms involved in the control of T_CM_ differentiation by Ang II have still to be determined.

Shao et al [18] showed the role of Ang II in T-helper differentiation. It was observed that Ang II induced an increase in INF-γ, characteristically produced by T_H_1 cells and a reduction in IL-4, the hallmark cytokine of T_H_2 lymphocytes through AT_1_ receptor. Similarly, we showed that Ang II has a crucial role in the induction of IFN-γ-producing T cells in the spleen of mice infected with *P. berghei* ANKA. In the same way, the systemic production of TNF-α and IFN-γ was significantly reduced in infected mice that received losartan treatment. Previous studies have shown that systemic IFN-γ is essential for the pathogenesis of experimental CM through the induction of TNF and ICAM-1 in brain endothelial cells culminating in leukocyte sequestration in the brain vasculature [61]. Also, IFN-γ signalling is important to increase macrophage infiltration, CD4^+^ and CD8^+^ T cells, to the brain of IFN-γ-receptor α chain deficient mice [62]. Although we found that **captopril** reduced significantly the sequestration of CD8^+^ T cells in the brain it did not interfere in INF-γ production.

Together with T_H_1 response modulation, the involvement of RAS in inducing the T_H_17 phenotype has been pointed out in autoimmune disease models, such as experimental autoimmune encephalomyelitis (EAE) [63]. In this case, AT_1_ blockage suppresses autoimmunity by reducing INF-γ and IL-17-producing T cells. In our results, besides inducing the T_H_1 phenotype, we observed that Ang II was also important to promote T_H_17 differentiation during *P. berghei* ANKA infection. It is known that IL-17 suppresses T_H_1 functions during inflammatory disease [64], but little is known about the functions of IL-17 during *Plasmodium* sp. infection. Here, we verified that both populations appeared concomitantly, indicating that IL-17 does not inhibit T_H_1 differentiation during malaria infection. These results are in agreement with a recent report that demonstrated that T_H_1 cells were normally induced in IL-17KO mice infected with *P. berghei* ANKA [65].

The induction of CD4^+^CD25^+^Foxp3^+^ regulatory T cells (Tregs) by malaria parasites has been described and is related to the ability of parasites to escape from both protective and harmful host immunity, especially concerning splenic T cells response [25]–[31]. Here, we observed that captopril and losartan treatments decreased CD4^+^CD25^+^Foxp3^+^ Tregs in mice infected with *P. berghei* ANKA as well as the levels of IL-10. In sharp contrast with our results, Platten et al [63] observed in EAE that the inhibition of ACE activity by lisinopril increases Tregs as well as IL-10 production. These differences could indicate that the effect of Ang II on the increase in CD4^+^CD25^+^Foxp3^+^ Tregs is specific to *P. berghei* ANKA infection.

It is postulated that TCR engagement and consequently, T cell activation, modulates the expression of the AT_1_ receptor of Ang II, making the cells more responsive to Ang II stimuli [17]. As we recently demonstrated using *in vitro* polyclonal stimuli, T cells activated by *P. berghei* ANKA antigens also showed higher levels of AT_1_ compared with naive cells. The upregulation of AT_1_ expression seems to be a positive loop dependent on the Ang II/AT_1_ axis, confirmed by the sensitivity to losartan treatment.

Our results support the notion that Ang II has a key role in the establishment of efficient splenic T cell activity during *P. berghei* ANKA infection. Therefore, we postulate that Ang II could be an important factor involved in a misbalanced parasite-induced T cell immune response in the spleen during *P. berghei* ANKA infection; this could be a crucial component in malaria pathogenesis.

## Supporting Information

Figure S1 Dose-response of Losartan (A) and Captopril (B) on the percentage of CD69^+^ spleen-derived T cells from *Plasmodium berghei* ANKA infected mice. Log (inhibitor) *vs.* response curve kinetics for the percentage of CD69^+^ T cells at day 6 post-infection were carried out using different daily doses of losartan or captopril (0.01–200 mg/Kg/d) to determine the IC_50_ values. Both losartan and captopril inhibited the induction of CD69^+^ T cells in the spleen of *P. berghei* ANKA infected mice. Experiment was performed in triplicate.
(TIF)

Figure S2 Losartan or captopril treatment inhibited *P. berghei* ANKA-induced splenic T cell activation. C57BL/6 mice were infected with *P. berghei* ANKA and treated with vehicle, losartan or captopril by gavage. T cells were isolated at day 6 post infection, stained with fluorescent antibodies and analyzed by flow cytometry. Absolute number of CD4+CD69+ T cells (A) and CD8+CD69+ T cells (B) observed in spleen. CD69 expression was analyzed by MIF on gated CD4+ (C) and CD8+ T cells (D). The results are expressed as means±SD. Statistically significant compared with values for *naive mice (*p*<0.05) and #vehicle-treated mice infected with *P. berghei* ANKA (*p*<0.05).
(TIF)

Figure S3 Influence of Ang II on T-cell sequestration to brain tissue and activation. (A) CFSE-labeled T cells were set in the lymphocyte region, determined in an FSC and SSC dot plot and confirmed by positive CD3– fluorescent staining. (B) The number of CD8+ T cells sequestered in the brain and (C) the percentage of CD8^+^CD69^+^ T cells was calculated. The results are expressed as the mean ± SD. Statistically significant compared with values for *naive mice (*p*<0.05) and #vehicle-treated mice infected with *P. berghei* ANKA (*p*<0.05).
(TIF)

Figure S4 Role of Ang II in CD62L/CD44 expression in spleen-derived T cells during *P. berghei* ANKA infection. C57BL/6 mice were infected with *P. berghei* ANKA and treated with vehicle, losartan or captopril by gavage. T cells were isolated at day 6 post infection, stained with fluorescent antibodies and analyzed by flow cytometry. (A) Representative dot plots of CD4^+^ and CD8^+^ naive or effector T cells obtained from gated CD3^+^ cells. The percentage of CD4^+^ or CD8^+^ CD62L^high^CD44^low^ (B, C), CD62L^high^CD44^high^ (D, E) and CD62L^low^CD44^high^ T cells (F, G) were calculated, respectively. The results are expressed as means±SE. Statistically significant compared with values for *naive mice (p<0.05) and #vehicle-treated mice infected with *P. berghei* ANKA (p<0.05).
(TIF)

Figure S5 Ang II is involved in the upregulation of CCR5 expression in splenic T cells during *P. berghei* ANKA infection. C57BL/6 mice were infected with *P. berghei* ANKA and treated with vehicle, losartan or captopril by gavage. CCR2 **(**A**)** and CCR5 **(**B**)** expression was analyzed by MIF on gated CD3^+^ T cells.
(TIF)

Figure S6 Cytokine production in naive mice and mice infected with *P. berghei* ANKA treated or not with losartan or captopril. TNF-α (A) and INF-γ (B) levels were determined by ELISA in the serum of naive mice and mice infected with *P. berghei* ANKA treated with vehicle, losartan or captopril, by gavage, at day 6 post infection. The results are expressed as means±SE. Statistically significant compared with values for *naive mice (*p*<0.05) and #vehicle-treated mice infected with *P. berghei* ANKA (*p*<0.05).
(TIF)
