# Supplementary figures and images for: Angiotensin II Is a New Component Involved in Splenic T Lymphocyte Responses during Plasmodium berghei ANKA Infection
Source: PLoS One. 2013 Apr 30;8(4):e62999. doi: 10.1371/journal.pone.0062999 (PMC3639972; doi:10.1371/journal.pone.0062999)

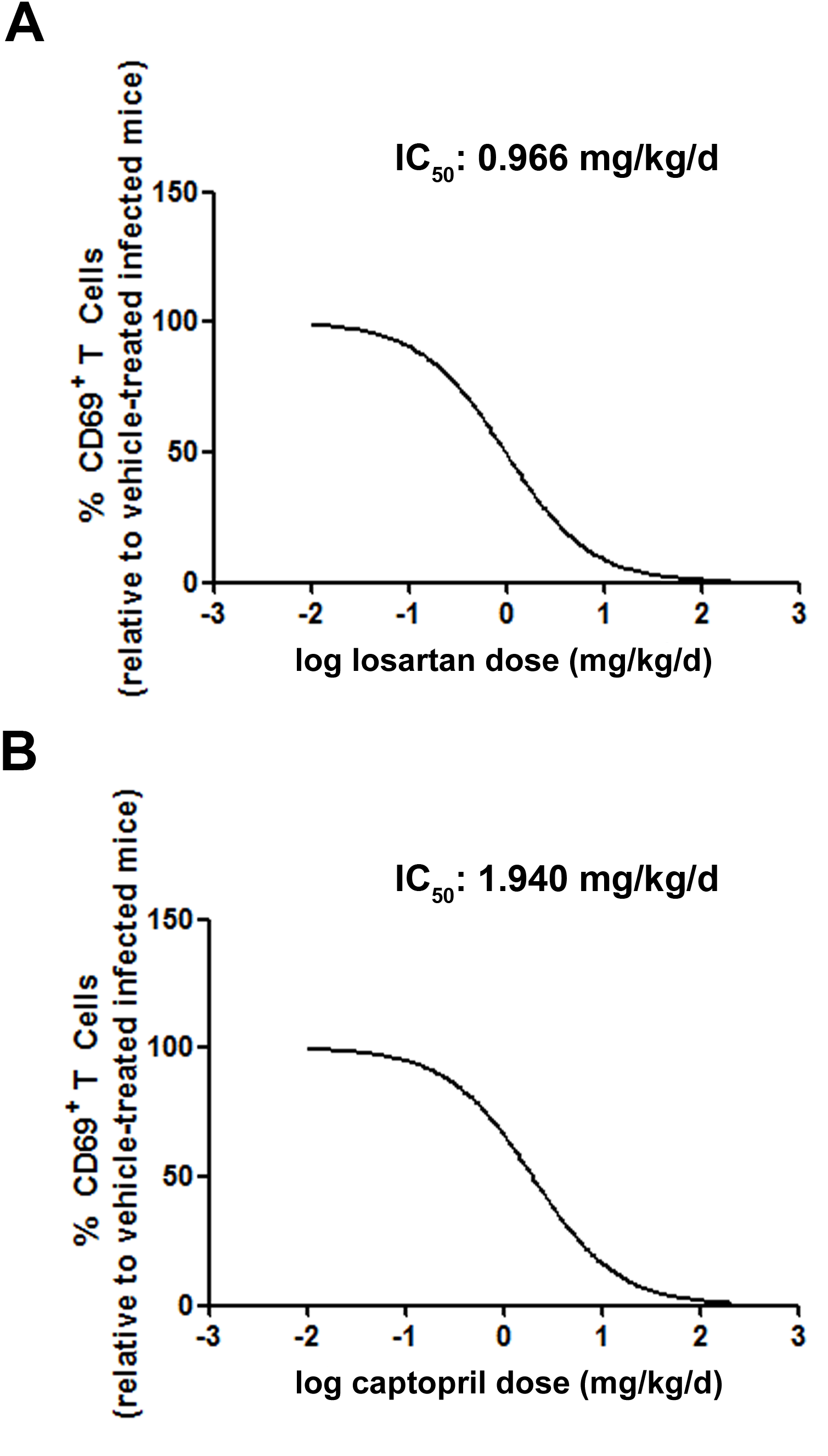

Supplement: Figure S1 — Dose-response of Losartan (A) and Captopril (B) on the percentage of CD69+ spleen-derived T cells from Plasmodium berghei ANKA infected mice. Log (inhibitor) vs. response curve kinetics for the percentage of CD69+ T cells at day 6 post-infection were carried out using different daily doses of losartan or captopril (0.01–200 mg/Kg/d) to determine the IC50 values. Both losartan and captopril inhibited the induction of CD69+ T cells in the spleen of P. berghei ANKA infected mice. Experiment was performed in triplicate. (TIF) [file pone.0062999.s001.tif]

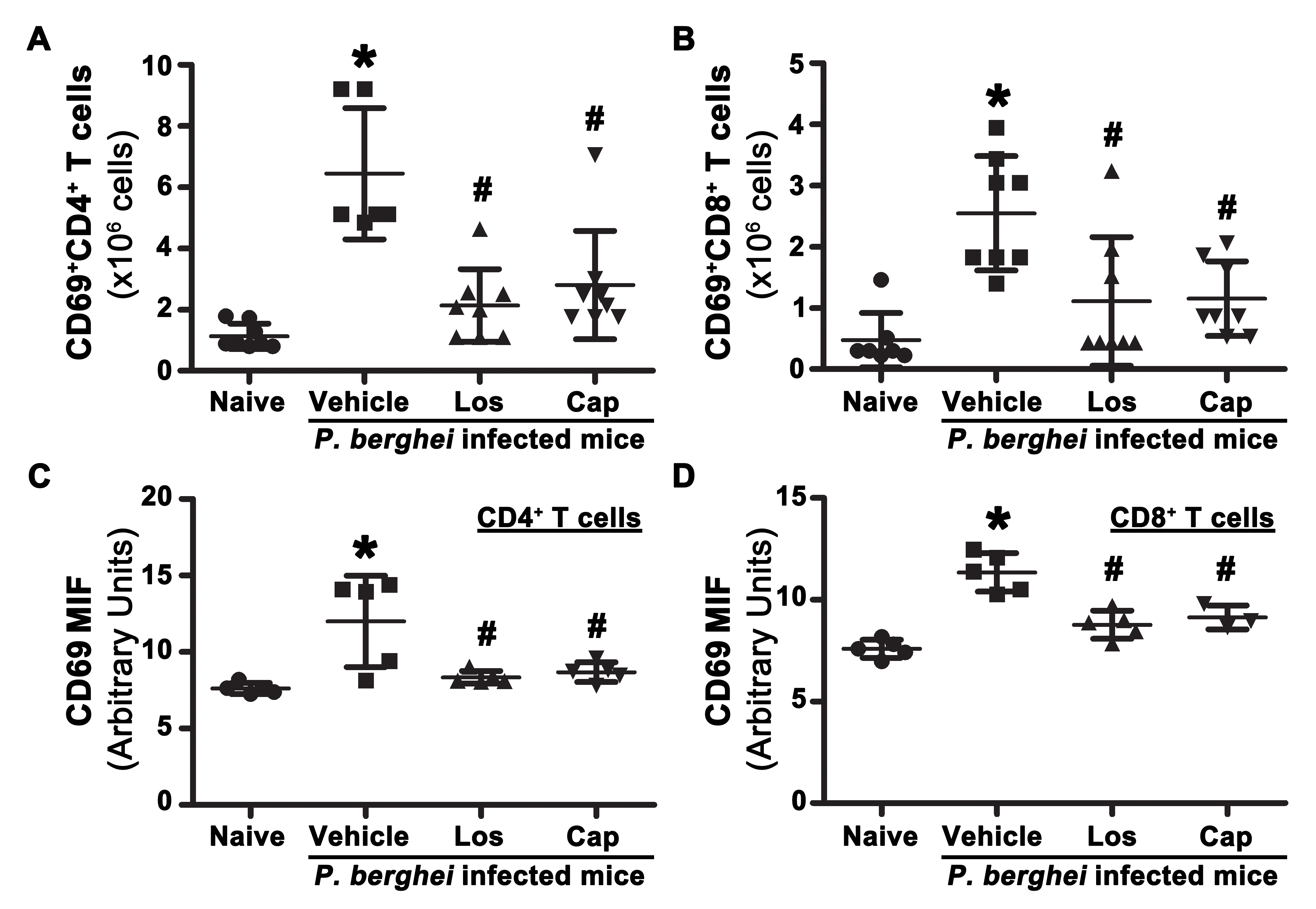

Supplement: Figure S2 — Losartan or captopril treatment inhibited P. berghei ANKA-induced splenic T cell activation. C57BL/6 mice were infected with P. berghei ANKA and treated with vehicle, losartan or captopril by gavage. T cells were isolated at day 6 post infection, stained with fluorescent antibodies and analyzed by flow cytometry. Absolute number of CD4+CD69+ T cells (A) and CD8+CD69+ T cells (B) observed in spleen. CD69 expression was analyzed by MIF on gated CD4+ (C) and CD8+ T cells (D). The results are expressed as means±SD. Statistically significant compared with values for *naive mice (p<0.05) and #vehicle-treated mice infected with P. berghei ANKA (p<0.05). (TIF) [file pone.0062999.s002.tif]

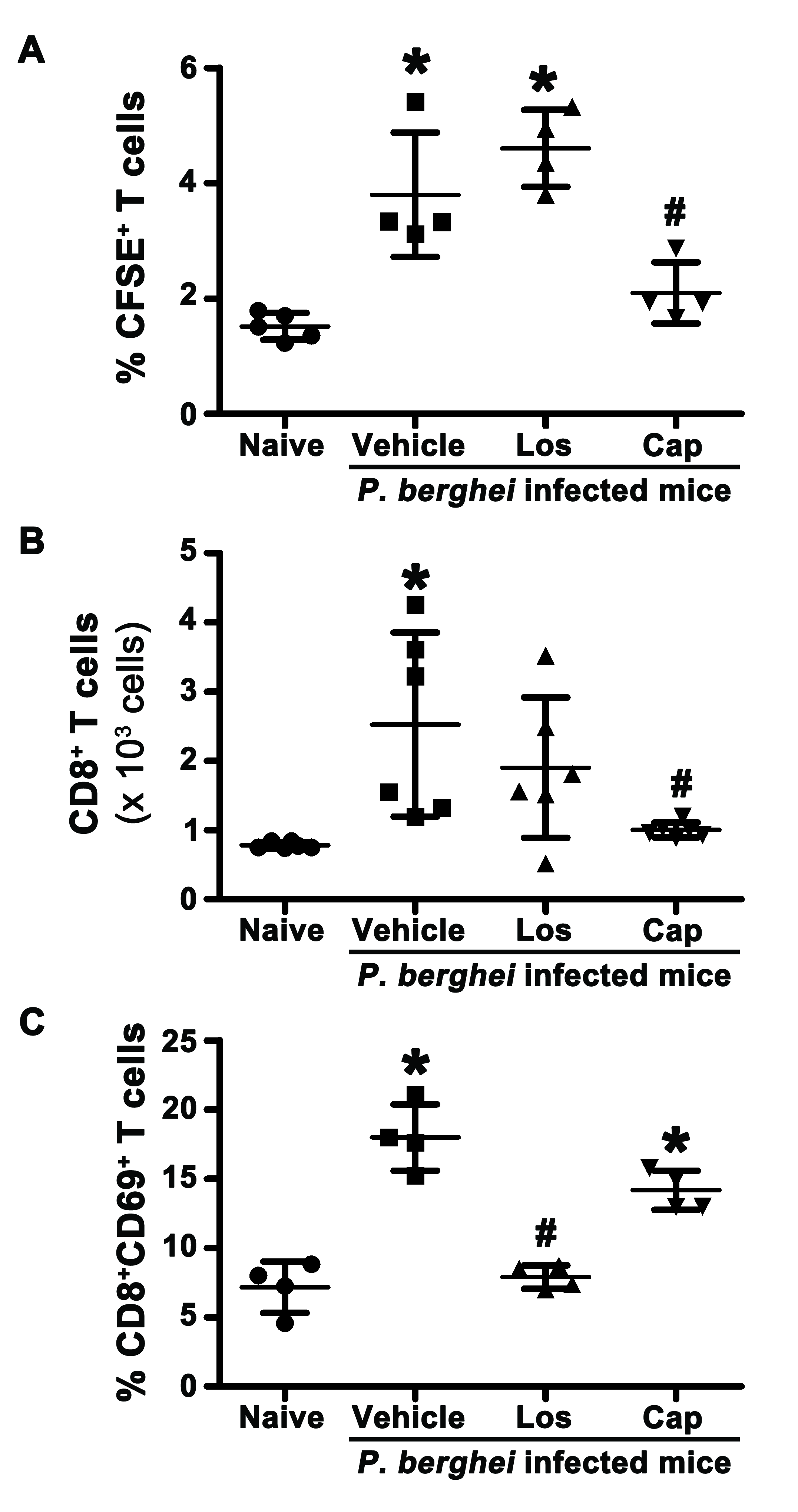

Supplement: Figure S3 — Influence of Ang II on T-cell sequestration to brain tissue and activation. (A) CFSE-labeled T cells were set in the lymphocyte region, determined in an FSC and SSC dot plot and confirmed by positive CD3– fluorescent staining. (B) The number of CD8+ T cells sequestered in the brain and (C) the percentage of CD8+CD69+ T cells was calculated. The results are expressed as the mean ± SD. Statistically significant compared with values for *naive mice (p<0.05) and #vehicle-treated mice infected with P. berghei ANKA (p<0.05). (TIF) [file pone.0062999.s003.tif]

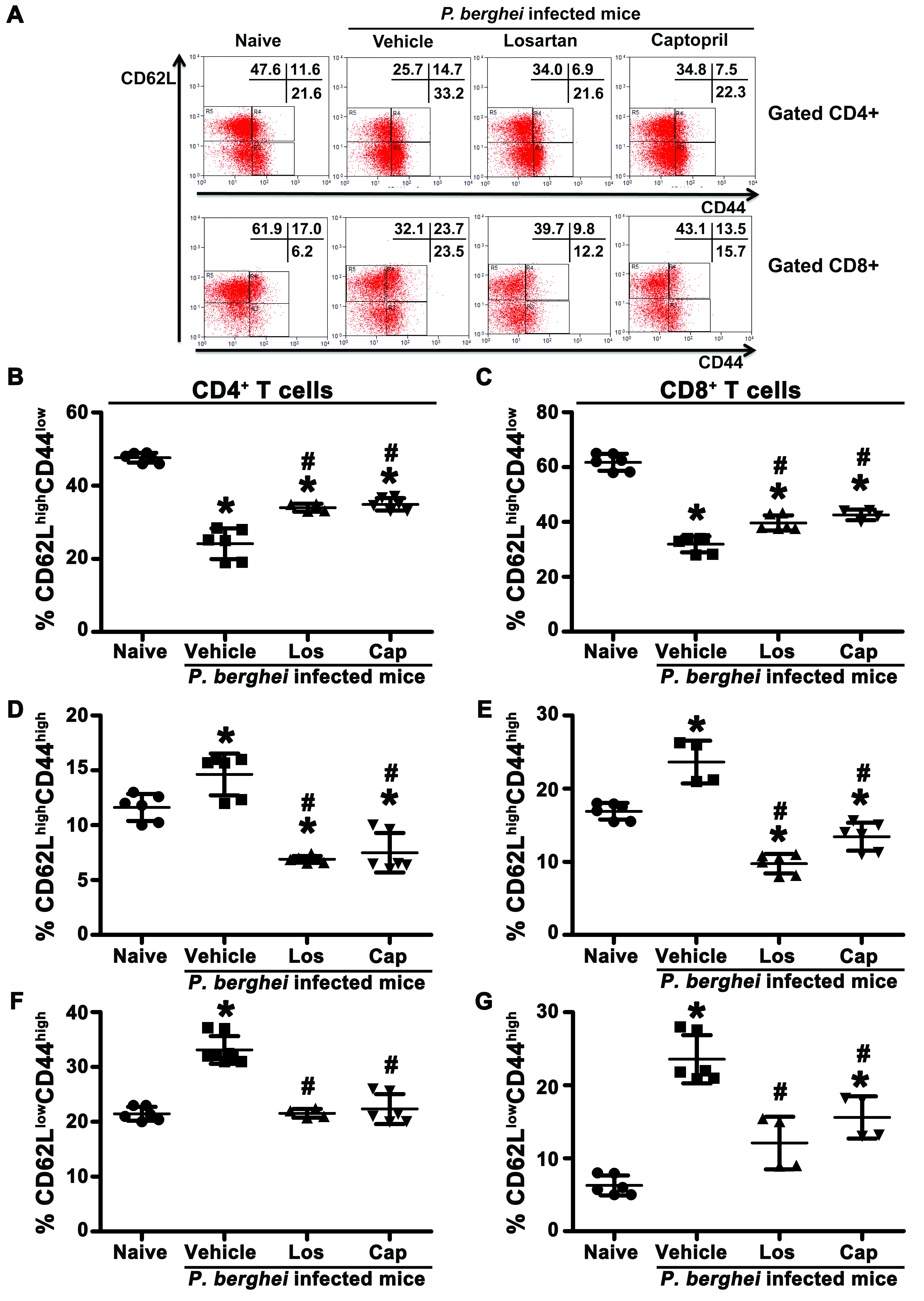

Supplement: Figure S4 — Role of Ang II in CD62L/CD44 expression in spleen-derived T cells during P. berghei ANKA infection. C57BL/6 mice were infected with P. berghei ANKA and treated with vehicle, losartan or captopril by gavage. T cells were isolated at day 6 post infection, stained with fluorescent antibodies and analyzed by flow cytometry. (A) Representative dot plots of CD4+ and CD8+ naive or effector T cells obtained from gated CD3+ cells. The percentage of CD4+ or CD8+ CD62LhighCD44low (B, C), CD62LhighCD44high (D, E) and CD62LlowCD44high T cells (F, G) were calculated, respectively. The results are expressed as means±SE. Statistically significant compared with values for *naive mice (p<0.05) and #vehicle-treated mice infected with P. berghei ANKA (p<0.05). (TIF) [file pone.0062999.s004.tif]

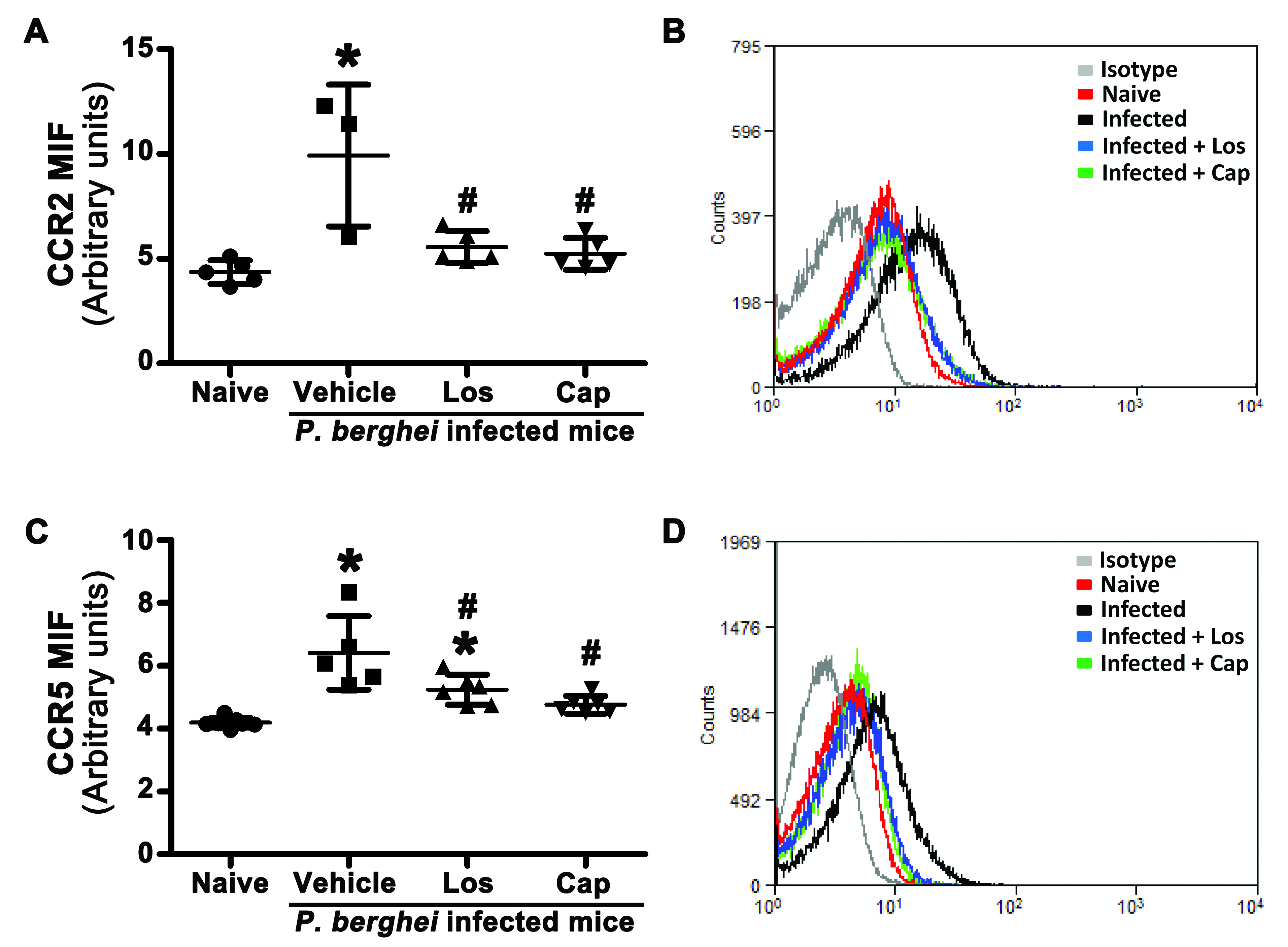

Supplement: Figure S5 — Ang II is involved in the upregulation of CCR5 expression in splenic T cells during P. berghei ANKA infection. C57BL/6 mice were infected with P. berghei ANKA and treated with vehicle, losartan or captopril by gavage. CCR2 (A) and CCR5 (B) expression was analyzed by MIF on gated CD3+ T cells. (TIF) [file pone.0062999.s005.tif]

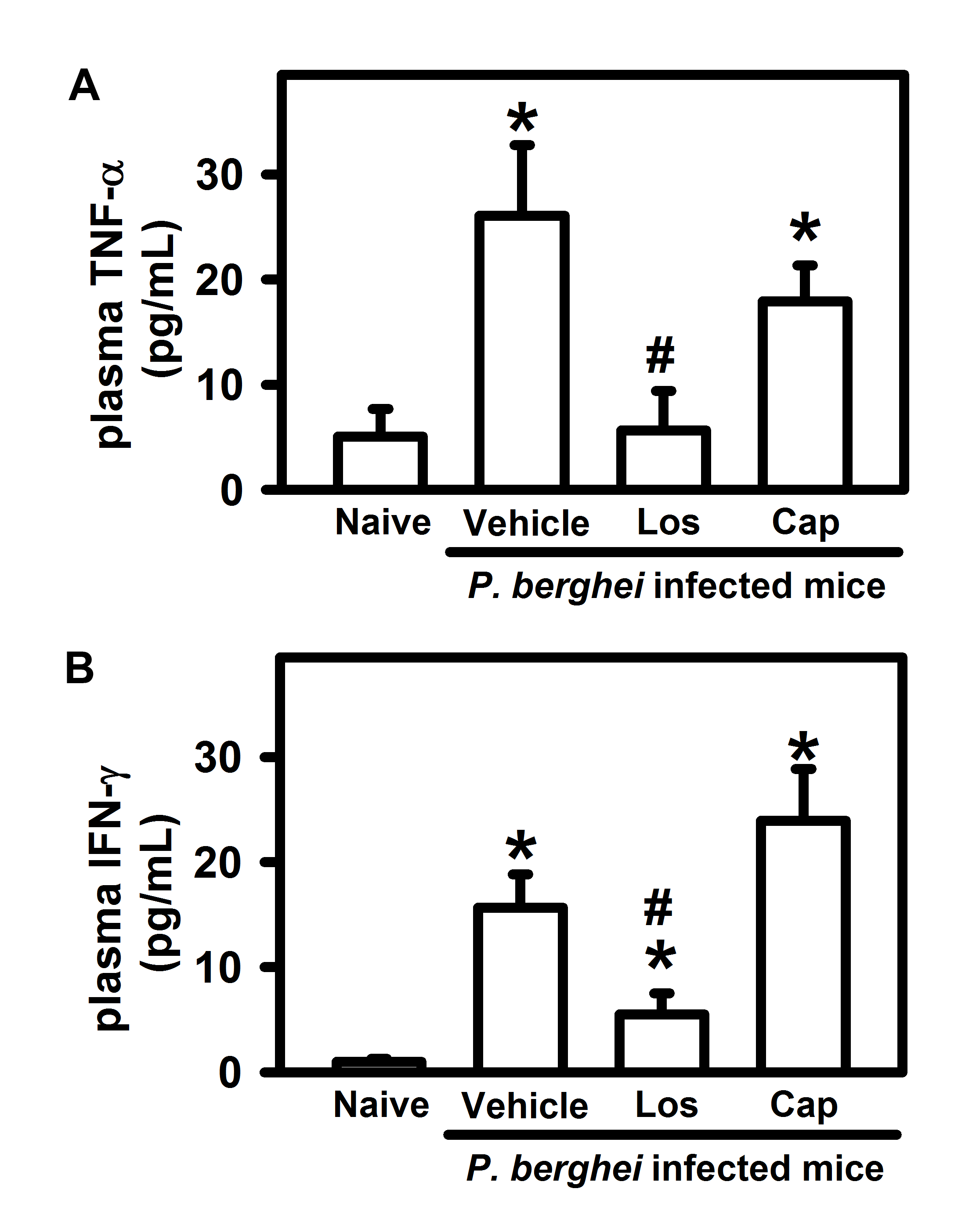

Supplement: Figure S6 — Cytokine production in naive mice and mice infected with P. berghei ANKA treated or not with losartan or captopril. TNF-α (A) and INF-γ (B) levels were determined by ELISA in the serum of naive mice and mice infected with P. berghei ANKA treated with vehicle, losartan or captopril, by gavage, at day 6 post infection. The results are expressed as means±SE. Statistically significant compared with values for *naive mice (p<0.05) and #vehicle-treated mice infected with P. berghei ANKA (p<0.05). (TIF) [file pone.0062999.s006.tif]
